# USP7 inactivation suppresses APC-mutant intestinal hyperproliferation and tumor development

**DOI:** 10.1016/j.stemcr.2022.12.013

**Published:** 2023-01-19

**Authors:** Laura Novellasdemunt, Anna Kucharska, Anna Baulies, Colin Hutton, Georgios Vlachogiannis, Dimitra Repana, Andrew Rowan, A. Suárez-Bonnet, Francesca Ciccarelli, Nicola Valeri, Vivian S.W. Li

**Affiliations:** 1 Stem Cell and Cancer Biology Laboratory, the Francis Crick Institute, 1 Midland Road, London NW1 1AT, UK; 2 Centre for Molecular Pathology, the Institute of Cancer Research, 15 Cotswold Road, Sutton, Surrey SM2 5NG, UK; 3 Cancer Systems Biology Laboratory, the Francis Crick Institute, 1 Midland Road, London NW1 1AT, UK; 4 Cancer Evolution and Genome Instability Laboratory, the Francis Crick Institute, 1 Midland Road, London NW1 1AT, UK; 5 Pathobiology and Population Sciences, Royal Veterinary College, Hatfield AL9 7TA, UK; 6 Experimental Histopathology, the Francis Crick Institute, 1 Midland Road, London NW1 1AT, UK

**Keywords:** colorectal cancer, Wnt signaling, USP7, APC, LGR5 stem cell

## Abstract

Adenomatous polyposis coli (APC) mutation is the hallmark of colorectal cancer (CRC), resulting in constitutive WNT activation. Despite decades of research, targeting WNT signaling in cancer remains challenging due to its on-target toxicity. We have previously shown that the deubiquitinating enzyme USP7 is a tumor-specific WNT activator in APC-truncated cells by deubiquitinating and stabilizing β-catenin, but its role in gut tumorigenesis is unknown. Here, we show *in vivo* that deletion of *Usp7* in *Apc*-truncated mice inhibits crypt hyperproliferation and intestinal tumor development. Loss of *Usp7* prolongs the survival of the sporadic intestinal tumor model. Genetic deletion, but not pharmacological inhibition, of *Usp7* in *Apc*^+/−^ intestine induces colitis and enteritis. USP7 inhibitor treatment suppresses growth of patient-derived cancer organoids carrying APC truncations *in vitro* and in xenografts. Our findings provide direct evidence that USP7 inhibition may offer a safe and efficacious tumor-specific therapy for both sporadic and germline *APC*-mutated CRC.

## Highlights

• Usp7 deletion in Apc-truncated mice reduces intestinal tumor development
• Intestine-specific loss of Usp7 is well tolerated in wild-type animals
• Loss of Usp7 prolongs the survival of the sporadic intestinal tumor model
• USP7 inhibitor suppresses growth of patient-derived organoids with APC mutations

## Graphical abstract

**Figure:**
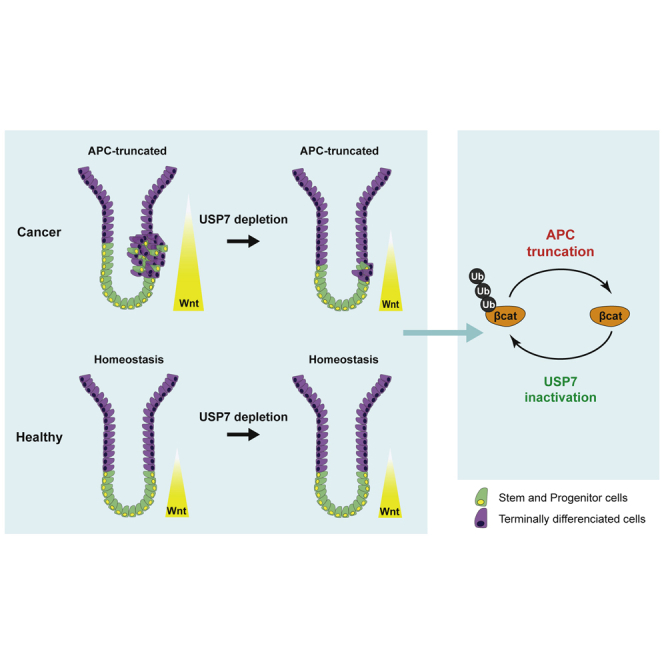

## Teaser

In this article, Li and colleague show that deletion of Usp7 in Apc-truncated mice inhibits crypt hyperproliferation and intestinal tumor development. Loss of Usp7 prolongs the survival of the sporadic intestinal tumor model and is well tolerated in healthy intestine. These findings provide direct evidence that USP7 could be a safe and efficacious tumor-specific target for APC-mutated colorectal cancer.

## Introduction

Colorectal cancer (CRC) is the third most commonly diagnosed cancer and the second cause of cancer-related death worldwide (Bray et al., 2018). CRC can be classified into two major categories: the microsatellite instable (MSI) subtype, characterized by defective mismatch repair machinery and hypermutations, and the non-hypermutated microsatellite stable (MSS) subtype. The majority of CRCs (∼85%) are MSS, characterized by sequential acquisition of genetic alterations including *APC*, *KRAS*, *TP53*, and *SMAD4* (Vogelstein and Kinzler 2004). Adenomatous polyposis coli (*APC*) mutation is the first step for tumor initiation in MSS CRC, leading to hyperactivation of the WNT signaling pathway (Nagase and Nakamura 1993; Cancer Genome Atlas Network 2012; Novellasdemunt et al., 2015). The canonical WNT/β-catenin pathway regulates the levels of the Wnt effector protein β-catenin through phosphorylation and ubiquitination-mediated degradation in the cytoplasmic β-catenin destruction complex. The latter consists of APC, AXIN, glycogen synthase kinase 3 (GSK3), and casein kinase 1 (CK1). In the absence of WNT ligands, β-catenin is sequentially phosphorylated by CK1 and GSK3, followed by recruitment of the E3 ubiquitin ligase β-TrCP to the destruction complex for ubiquitination and subsequent proteasomal degradation (Aberle et al., 1997; Kitagawa et al., 1999; Liu et al., 2002). Engagement of WNT ligands to the receptors inhibits the β-catenin destruction complex, leading to accumulation of β-catenin in the cytoplasm and nucleus for transcriptional activation of the WNT target genes (Li et al., 2012).

Previous studies showed that deletion of *Apc* in mice leads to crypt hyperproliferation and adenoma formation in the intestine through constitutive activation of WNT signaling and β-catenin/T cell factor (TCF) transcription of target genes (Shibata et al., 1997; Sansom et al., 2004). In human, somatic and germline mutations of *APC* were discovered in patients with CRC in 1991 (Groden et al., 1991; Joslyn et al., 1991; Kinzler et al., 1991; Nishisho et al., 1991), where the majority of somatic *APC* mutations occur in the “mutation cluster region” (MCR) between codons 1286 and 1513 (Polakis, 1995; Vogelstein and Kinzler 2004). Region-specific *APC* mutations have been associated with differential WNT/β-catenin transcriptional activity and tumor susceptibility (Gaspar and Fodde 2004). We have previously shown that *APC*-truncating mutations activate WNT signaling through abrogation of β-catenin ubiquitination within the destruction complex in CRCs (Li et al., 2012). Despite decades of research, safe drugs that correct *APC* loss in (colon) cancer remain elusive.

A number of inhibitors targeting upstream components of the WNT pathway (e.g., the WNT receptors Frizzled [DeAlmeida et al., 2007; Gurney et al., 2012; Jimeno et al., 2017; Moore et al., 2019] and the WNT ligand secretory machinery: porcupine (Jung and Park 2020]) are currently in clinical trials. However, these inhibitors are not effective in *APC*-mutated CRC where constitutive WNT activation is independent of ligand-receptor activation. Tankyrase inhibitors and β-catenin/CPB (cAMP response element-binding protein) antagonists acting in the cytoplasm and nucleus, respectively, have been previously proposed to suppress WNT signaling in *APC*-mutated CRC cells (Chen et al., 2009; Huang et al., 2009; Waaler et al. 2011, 2012; Andrew et al., 2016; Mariotti et al., 2017). However, these inhibitors exhibit high on-target toxicity due to the pivotal role of WNT signaling in other healthy tissues (Lau et al., 2013). This poses safety concerns for the use of WNT inhibitors in the clinic. For many years, the WNT pathway has been considered undruggable.

We have recently found that a deubiquitinating enzyme USP7 is responsible for WNT activation, specifically in *APC*-mutated CRC (Novellasdemunt et al., 2017). We showed that USP7 is recruited to the APC-truncated destruction complex for β-catenin deubiquitination and stabilization. Deletion of USP7 in cancer cell lines and organoids inhibits WNT activation by restoring β-catenin ubiquitination specifically in *APC*-mutated, but not wild-type (WT), cells, suggesting that USP7 may represent a tumor-specific drug target. Contrary to our finding, a more recent study showed that USP7 is a potent negative regulator of WNT/β-catenin signaling by deubiquitinating and stabilizing AXIN (Ji et al., 2019), casting doubt on the use of USP7 as WNT inhibitor for patients with *APC*-mutated CRC. Importantly, the functional role of USP7 in CRC has not yet been explored *in vivo*.

Here, we investigate the role of USP7 in tumor development and intestinal homeostasis *in vivo*. Comprehensive analyses of three different *Apc*-deletion mouse models show that loss of *Usp7* significantly reduces crypt hyperproliferation, WNT activation, and tumor development in *Apc*-deficient intestine. Consistent with our previous observation in cancer cell lines and organoids, deletion of *Usp7* in the *Apc* WT animals does not perturb intestinal homeostasis. We further validate the findings in human CRC, where treatment with a USP7 inhibitor suppresses growth in both patient-derived organoids (PDOs) and xenografts carrying APC-truncating mutations, supporting the notion that USP7 can be used as a tumor-specific drug target for *APC*-mutated CRCs by suppressing WNT signaling.

## Results

### *Usp7* depletion suppresses intestinal crypt hyperproliferation and WNT activation mediated by homozygous loss of *Apc*

**Figure 1:**
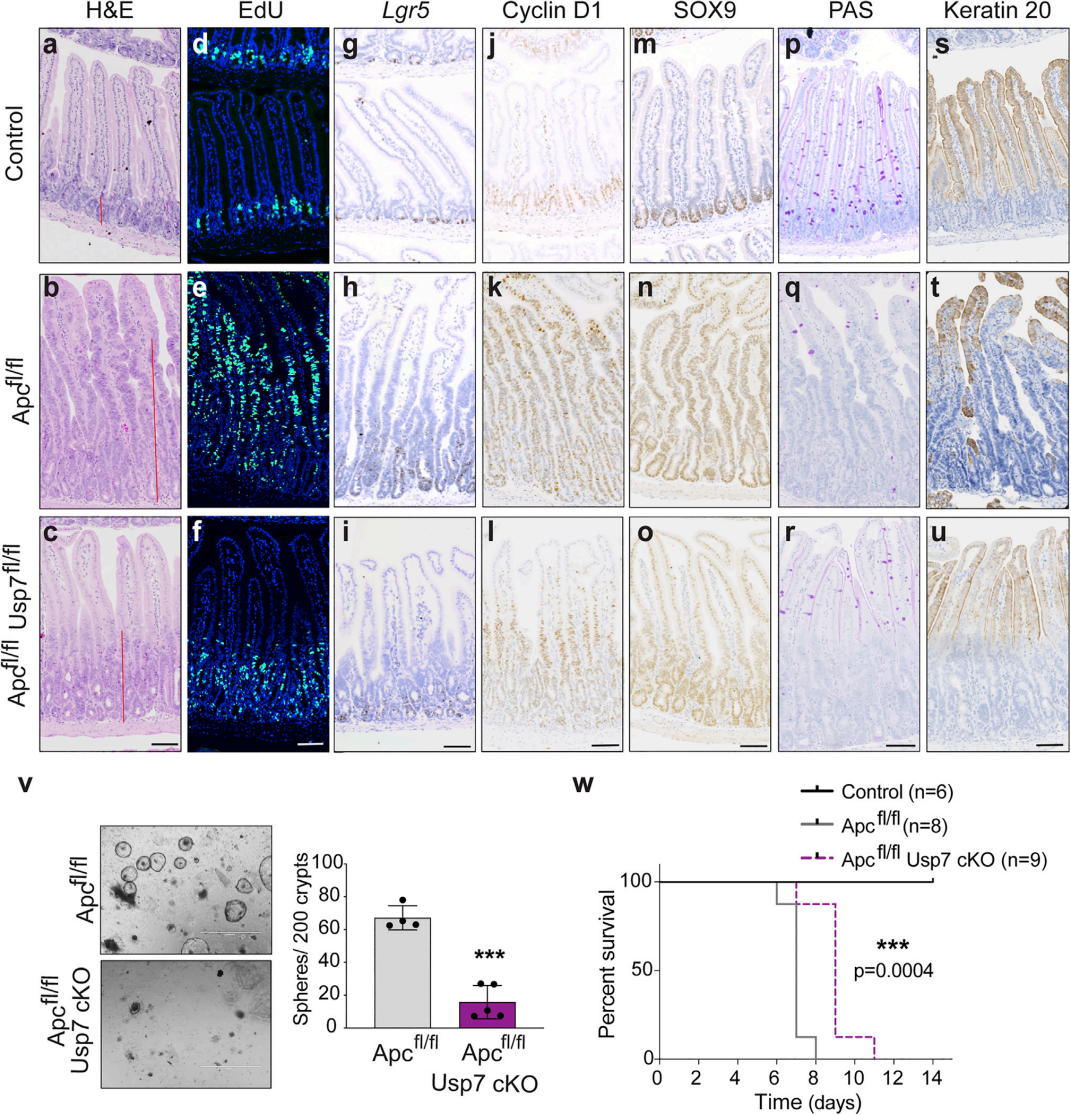
Loss of *Usp7* ameliorates acute WNT activation phenotype (A–U) Histology and immunostaining using the indicated markers (n = 3 biological replicates per group). Representative images of H&E staining (A–C); EdU (D–F); *Lgr5* RNAscope (G–I); cyclin D1 (J–L); SOX9 (M−O); PAS (P–R); and keratin 20 staining (S–U) from Villin^CreERT2^ (control), Villin^CreERT2^;Apc^fl/fl^ (Apc^fl/fl^), and *Villin*^CreERT2^;*Apc*^fl/fl^;*Usp7*^fl/fl^ (*Apc*^fl/fl^*Usp7*^fl/fl^) intestine at 6 dpi. Scale bars, 50 μm. (V) Representative images of colony-formation assay of organoids derived from *Apc*^fl/fl^ and *Apc*^fl/fl^*Usp7*^fl/fl^ intestines at 6 dpi. Scale bar, 1,000 μm. Right, quantitation of the number of spheres that form after seeding 200 crypts. Data are mean ± SE. At least n = 4 mice as organoid donors per group. p values were determined using the unpaired two-sided t test (^∗∗∗^p < 0.001). (W) Kaplan-Meier survival analysis of control, *Apc*^fl/fl^, and *Apc*^*f*l/fl^*Usp7*^fl/fl^ mice. p values were determined using the Mantel-Cox test.

To explore the therapeutic role of USP7 in CRC, we first examined the effect of *Usp7* deletion in an acute WNT activation model mediated by homozygous loss of *Apc*. *Villin*^CreERT2^;*Apc*^fl/fl^ (*Apc*^fl/fl^) mice (Shibata et al., 1997) were crossed with *Villin*^CreERT2^;*Usp7*^fl/fl^ mice (Kon et al., 2011) to obtain intestinal-specific conditional deletion of *Apc* and *Usp7*. Homozygous loss of *Apc* caused hyperproliferation of the intestinal crypts within 6 days after tamoxifen induction (Figures 1A, 1B, 1D, and 1E). This was accompanied by increased expression of the stem cell markers *Lgr5* (Barker et al., 2007) and CD44 (Figures 1G, 1H, and S1A) and WNT targets cyclin D1 and SOX9 (Figures 1J, 1K, 1M, and 1N) compared with WT control (*Villin*^CreERT2^) intestine and by loss of differentiation markers as indicated by periodic acid Schiff (PAS) staining (goblet cells) (Figures 1P and 1Q) and keratin 20 (Figures 1S and 1T). Strikingly, deletion of *Usp7* in *Apc*^fl/fl^ intestine (*Apc*^fl/fl^ *Usp7*^fl/fl^) resulted in a marked suppression of crypt hyperproliferation (Figures 1C, 1F, and S1B) and a significant reduction in the size of the crypts (Figure S1C) and the length of the crypt-villus axis (Figure S1D) throughout the gut. In addition, the stem cell markers *Lgr5* (Figure 1I) and CD44 (Figure S1A) and WNT targets cyclin D1 and SOX9 (Figures 1L and 1O) were consistently suppressed in the *Apc*^fl/fl^ *Usp7*^fl/fl^ intestine, while differentiation was partially restored (Figures 1R, 1U, and S1E).

To validate the WNT inhibition and growth suppression phenotype, we further examined the intestinal organoids derived from *Apc*^fl/fl^ and *Apc*^fl/fl^ *Usp7*^fl/fl^ animals. Consistent with the *in vivo* data, *Usp7* depletion strongly suppressed the organoid formation efficiency in the *Apc*-deficient background (Figure 1V). Quantitative RT-PCR analysis confirmed the loss of *Usp7* transcripts in organoids (Figure S1F), where WNT target genes (*Axin2* and *Sox9*) and stem cell markers (*Olfm4* and *Ascl2*) were significantly downregulated in the *Apc*^fl/fl^ *Usp7*^fl/fl^ organoids (Figure S1F). Together, the results indicate that *Usp7* deletion in the intestine suppresses WNT signaling and crypt hyperproliferation mediated by *Apc* mutation.

Interestingly, deletion of *Usp7* prolonged the survival of the *Apc*^fl/fl^ mice for an average of 7–9 days but not beyond (Figure 1W). This is likely due to the highly aggressive phenotype of the acute WNT activation model driven by homozygous loss of *Apc*, where the entire gut epithelium is acutely transformed into adenomatous tissue.

### *Usp7* is dispensable for intestinal homeostasis in WT mice

**Figure 2:**
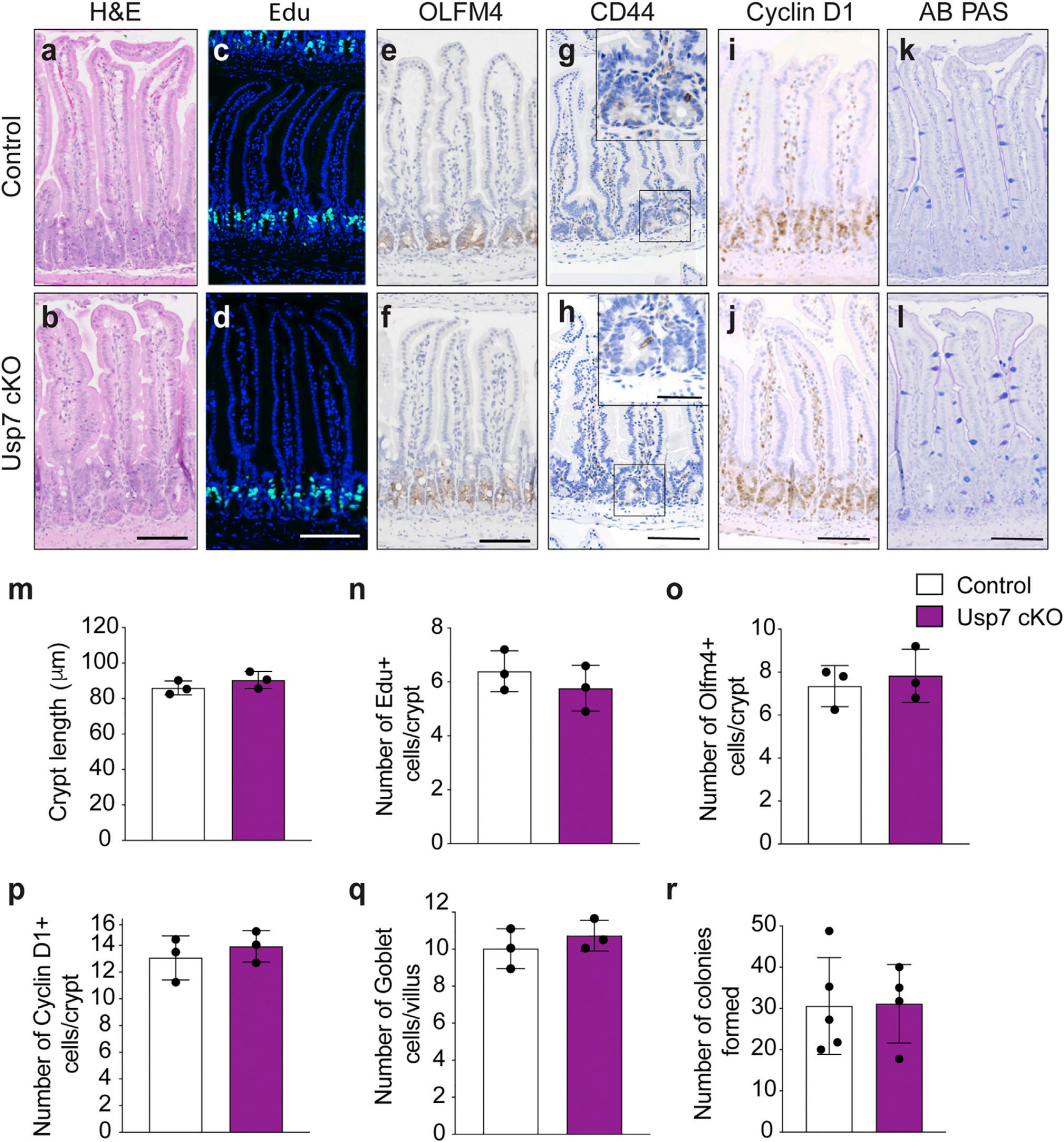
*Usp7* inhibition in wild-type mice does not perturb intestinal homeostasis (A–J) Histology and immunostaining using the indicated markers. n = 3 biological replicates per group. Representative images of H&E staining (A and B); EdU (C and D); OLFM4 (E and F); CD44 (G and H); cyclin D1 (I and J); and Alcian blue (AB)-PAS (K and L) from *Villin*^CreERT2^ (control) and *Villin*^CreERT2^;*Usp7*^fl/fl^ (*Usp7* cKO) intestine at 17 dpi. High-magnification images are shown in the insets. Scale bars, 50 μm. (M) Quantitation of crypt length (μm) from (A) and (B). Each dot represents an average of at least 20 crypts per animal. Data are mean ± SE. n = 3 biological replicates per group. (N–Q) Quantitation of EdU-positive cells (N) from (C) and (D); OLFM4 (O) from (E) and (F); cyclin D1 (P) from (I) and (J); and AB-PAS staining (indicative of goblet cells) (Q) from (K) and (L). Each dot represents an average of at least 20 crypts per animal (except for EdU, which is 10 crypts per animal). Data are mean ± SE. n = 3 biological replicates per group. p values were determined using the unpaired two-sided t test (^∗^p < 0.05). (R) Quantitation of the organoid-formation assay in organoids derived from control and *Usp7* cKO mice. Data are mean ± SE. Control, n = 5; *Usp7* cKO, n = 3.

Our previous data using CRC cell lines and organoids showed that USP7 is essential for pathological WNT activation in APC mutant cells but not physiological WNT signaling in normal cells, suggesting that USP7 can be used as tumor-specific drug target (Novellasdemunt et al., 2017). However, an independent study showed that USP7 functions as a negative regulator of WNT, where inhibition of USP7 increases WNT signaling in normal cells (Ji et al., 2019). To clarify the role of USP7 in normal intestine, we generated *Villin*^CreERT2^;*Usp7*^fl/fl^ (*Usp7* conditional knockout [cKO]) mice to induce *Usp7* deletion specifically in the intestine upon tamoxifen administration. *Usp7*-depleted intestine did not show any noticeable histological differences, including crypt length (Figures 2A, 2B, and 2M) or cell proliferation (Figures 2C, 2D, and 2N) 17 days post-induction (dpi). Similarly, expression of the stem cell markers OLFM4 (Figures 2E, 2F, and 2O) and CD44 (Figures 2G and 2H), WNT target cyclin D1 (Figures 2I, 2J, and 2P), and goblet cell differentiation (Figures 2K, 2L, and 2Q) were unaltered. We further examined if p53 is activated upon *Usp7* deletion since it is also a proposed target of USP7 (Li et al., 2002; Meulmeester et al., 2005). Immunostaining of the *Usp7* cKO intestine did not show any induction of p21 expression (Figure S2A), indicating that *Usp7* loss does not activate p53-mediated growth arrest in the intestine.

Next, we established organoids from the control and *Usp7* cKO intestine, which showed no differences in morphology (Figure S2B) or organoid formation efficiency (Figure 2R), suggesting that *Usp7* deficiency does not affect intestinal stem cell proliferation. Indeed, unlike in the *Apc*^fl/fl^ background, loss of *Usp7* in WT organoids did not alter the expression of stem cells, WNT target genes, or differentiation markers (Figures S2C–S2F). The results demonstrate that deletion of *Usp7* alone does not alter WNT signaling or tissue homeostasis in normal WT intestine.

To further study the long-term effect of *Usp7* deletion, *Usp7* cKO animals were aged for 1.5 years, followed by tissue analysis. Consistent with the short-term deletion data, long-term loss of *Usp7* did not cause any significant changes in tissue morphology (Figures S2G and S2H), proliferation (Figures S2I and S2J), and expression of stem cell markers (Figures S2K, S2l, S2Q, and S2R) and WNT target genes (Figures S2M–S2P). Quantitative RT-PCR analysis of the intestinal crypts isolated from these mice further confirmed that the stem cell markers and WNT target gene expression remained unchanged after long-term deletion of *Usp7* (Figures S2S–S2U). Our data support the notion that loss of *Usp7* alone in WT mice does not impact WNT signaling or intestinal stem cell proliferation.

### *Usp7* depletion suppresses intestinal tumor development in both germline and sporadic *Apc* mutant mice

**Figure 3:**
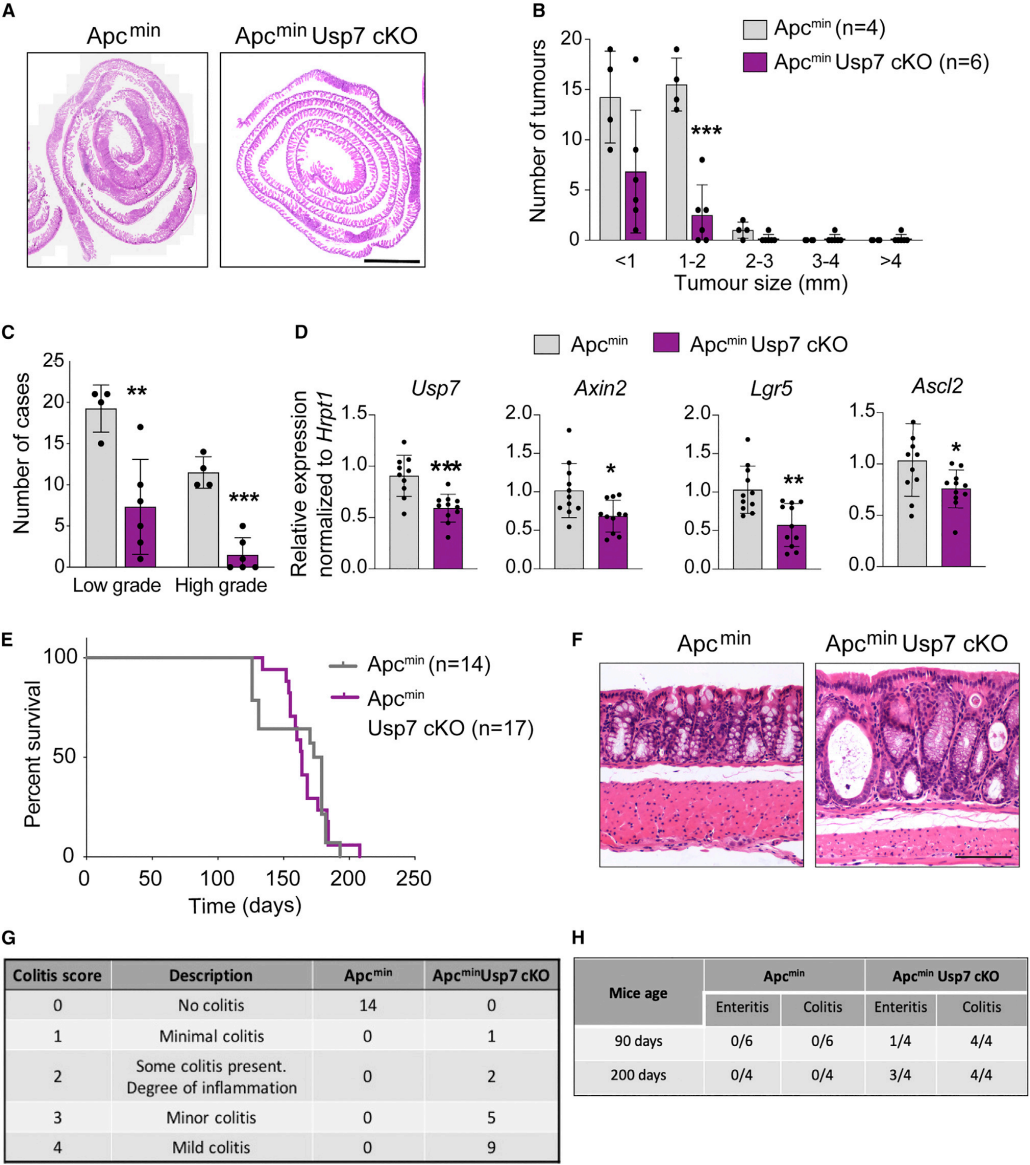
Loss of *Usp7* reduces tumor number in *Apc*^min^ mice (A) Representative H&E stainings of the small intestines of the indicated genotypes. Scale bar, 2000 μm. (B) Total number of adenomas 120 days after induced *Usp7* loss. Data are mean ± SE. *Apc*^min^ control n = 4 and *Apc*^min^*Usp7* cKO n = 6. (C) Quantitation of the grades of the adenomas that developed in the indicated mice. Data are mean ± SE. *Apc*^min^ control n = 4 and *Apc*^min^*Usp7* cKO n = 6 biological replicates. (D) mRNA expression of the indicated genes was analyzed by qRT-PCR in tumors isolated from mice with the indicated genotypes (n = 3 biological replicates per condition). Data are presented as fold change normalized to *hrpt1* control. Error bars represent ± SE. (E) Kaplan-Meier survival analysis of control *Apc*^min^ and *Apc*^min^*Usp7* cKO mice. p value was determined using the Mantel-Cox test. (F) Representative H&E staining from *Apc*^min^ control and *Apc*^min^*Usp7* cKO mice showing colitis present in the *Apc*^min^*Usp7* cKO intestine. Scale bar, 100 μm. (G) Table summarizing the number of mice that developed colitis in the indicated genotypes. (H) Table depicting the number of mice that developed colitis and enteritis in the indicated genotypes and time. Short term refers at around 90 dpi and long term refers at around 200 dpi. All mice showing enteritis were of grade 2–3 (grading description in the experimental procedures).

Next, we investigated the role of *Usp7* in intestinal tumorigenesis. The *Apc*^min^ mouse strain, modeling human familial adenomatous polyposis (FAP), was used as a chronic spontaneous model for intestinal tumor development (Su et al., 1992). Comparison of *Apc*^min^ and *Apc*^min^ *Usp7* cKO mice showed that *Usp7* deletion significantly reduced tumor numbers at 4 months of age (Figures 3A and 3B). Histological analysis revealed that both low- and high-grade dysplasias were present in all *Apc*^min^ control animals, whereas the majority of the adenomas in the *Apc*^min^ *Usp7* cKO mice were low-grade dysplasias (Figure 3C). The results indicate that *Usp7* deficiency inhibits tumor progression in *Apc*^min^ mice. Loss of *Usp7* was confirmed by quantitative RT-PCR analysis of the isolated crypts of the animals (Figure S3A). Immunostaining of cleaved caspase3 did not show any difference between *Apc*^min^ control and *Apc*^min^ *Usp7* cKO tumors, indicating that the tumor reduction upon *Usp7* deletion was not caused by increased apoptosis (Figure S3B). Quantitative RT-PCR analysis of the isolated tumors showed that expression of the WNT target genes and stem cell markers *Lgr5*, *Ascl2*, and *Axin2* were suppressed in the *Usp7* cKO tumors (Figure 3D), suggesting that *Usp7* depletion inhibits WNT signaling in the *Apc*-deficient intestinal tumors. Interestingly, histological analysis of the established tumors in both *Apc*^min^ and *Apc*^min^ *Usp7* cKO mice showed reduced proliferation as assessed by 5-ethynyl-2′-deoxyuridine (EdU) staining (Figures S3C and S3D) and decreased stem cell marker SOX9 expression (Figures S3E and S3F). Surprisingly, unlike our observations at earlier time points, we observed no difference in the survival of *Apc*^min^ *Usp7* cKO mice or their overall tumor burden compared with *Apc*^min^ control (Figures 3E and S3G). It is worth noting that the *Apc*^min^ *Usp7* cKO tumors showed incomplete depletion of *Usp7* (Figure 3D), suggesting some degree of escape phenotype in these tumors. Importantly, histological examination of the intestinal tissues revealed that all *Apc*^min^ *Usp7* cKO animals displayed various degrees of colitis pathology (Figures 3F and 3G) as well as enteritis (Figures 3H and S3H), which may have worsened the health of the animals. This was confirmed by the enrichment of CD68 staining of macrophages/monocytes in *Apc*^min^ *Usp7* cKO mice at both 90- and 200-day-old mice, indicating the involvement of the innate immune system in this process (Figure S3I). The data suggest that although loss of *Usp7* does not alter tissue homeostasis in WT intestine, there might be a deleterious effect when *Usp7* is inhibited in intestinal cells carrying only one copy of WT *Apc* gene (as in the *Apc*^min^ mouse model) due to haplo-insufficiency. We speculate that the colitis and enteritis observed in the *Apc*^min^ *Usp7* cKO intestine may promote inflammation-associated CRC development over time (Grivennikov 2013), which may compensate for the tumor-suppressive effect of *Usp7* loss in these animals.

**Figure 4:**
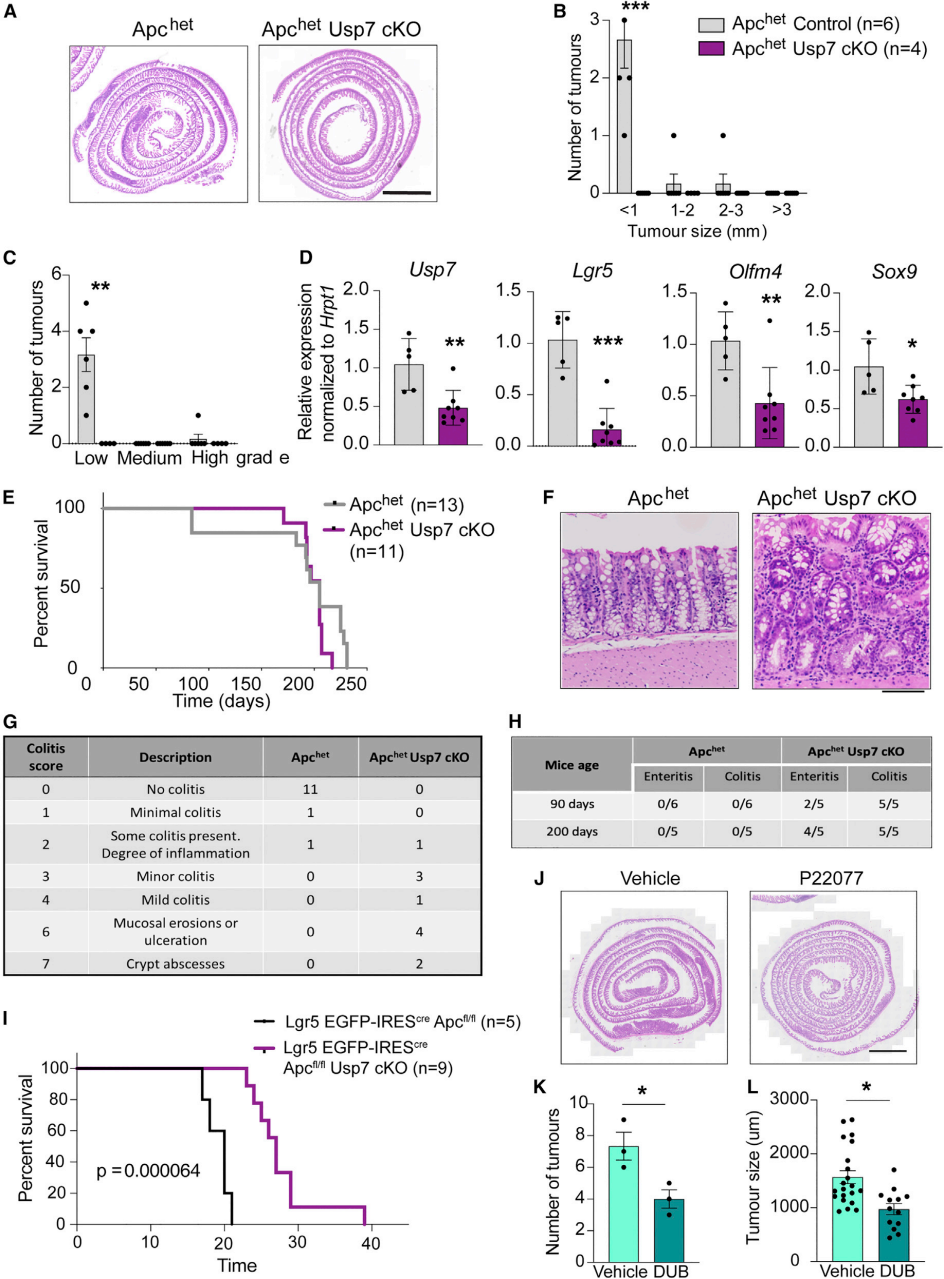
*Usp7* deletion inhibits tumor growth in *Apc*^het^ mice (A) Representative H&E stainings of the small intestines of the indicated genotypes. Scale bar, 2000 μm. (B) Total number of adenomas 84 days after induced *Usp7* loss. Data are mean ± SE. *Apc*^het^ control n = 6 and *Apc*^het^*Usp7* cKO n = 4 biological replicates. Error bars represent ±SE. p values were determined using the unpaired two-sided t test (^∗∗∗^p < 0.001). (C) Quantitation of the grades of the adenomas that developed in the indicated mice. Data are mean ± SE. *Apc*^het^ control n = 6 and *Apc*^het^*Usp7* cKO n = 4. Error bars represent ± SE. p values were determined using the unpaired two-sided t test (^∗∗^p < 0.01). (D) mRNA expression of the indicated genes was analyzed by qRT-PCR in tumors isolated from mice with the indicated genotypes (n = 3 biological replicates per condition). Data are presented as fold change normalized to *hrpt1* control. Error bars represent ± SE. (E) Kaplan-Meier survival analysis of control, *Apc*^het^, and *Apc*^het^*Usp7* cKO mice. p values were determined using the Mantel-Cox test. (F) Representative H&E staining from *Apc*^het^ control and *Apc*^het^*Usp7* cKO mice showing colitis present in the *Apc*^het^*Usp7* cKO mice. Scale bar, 100 μm. (G) Table summarizing the number of mice that developed colitis in the indicated genotypes. (H) Table summarizing the number of mice that developed colitis and enteritis in the indicated genotypes and time. Short term refers to at around 90 dpi and long term refers to at around 200 dpi. All mice showing enteritis were of grade 2–3 (grading description in experimental procedures). (I) Kaplan-Meier survival analysis of *Lgr5* EGFP-IRES^cre^*Apc*^f/fl^ (control) and *Lgr5* EGFP-IRES^cre^*Apc*^f/fl^*Usp7* cKO mice. p values were determined using the Mantel-Cox test (^∗∗∗^p < 0.001). (J) Representative H&E stainings of the small intestines of the indicated genotypes. Scale bar, 2000 μm. (K and L) Number of tumors (K) and tumor size (L) developed by *Apc*^min^ treated with P22077 or vehicle (DMSO) for 21 days (n = 3 biological replicates per condition). Error bars represent ± SE. p values were determined using the unpaired two-sided t test (^∗^p < 0.05).

To validate the hypothesis of *Apc* haplo-insufficiency in *Usp7* KO background, we generated another *Apc* heterozygous model using *Villin*^CreERT2^;*Apc*^fl/+^ (*Apc*^het^) animals and further crossed to *Usp7* cKO mice. Tamoxifen was injected to the *Apc*^het^ and *Apc*^het^ *Usp7* cKO animals, and intestinal tissues were harvested at around 90 dpi for histological analysis. Remarkably, adenomas were observed in all 6 *Apc*^het^ mice, while none of the *Apc*^het^ *Usp7* cKO mice developed any tumor (Figures 4A–4C). This is consistent with our earlier observation that loss of *Usp7* inhibits *Apc*-deficient tumor development. Quantitative RT-PCR analysis confirmed *Usp7* depletion as well as reduced expression of stem cell markers (*Lgr5* and *Olfm4*) and WNT target gene (*Sox9*) in the *Apc*^het^ *Usp7* cKO intestine (Figure 4D). Immunostaining showed aberrant expression of SOX9, cyclin D1, and EdU in the *Apc*^het^ tumors (Figures S4A, S4C, and S4E), while expression of the WNT targets and proliferation markers remained restricted to the untransformed crypts of the *Apc*^het^ *Usp7* cKO intestine (Figures S4B, S4D, and S4F). Similar to the *Apc*^min^ model, we observed no difference in the survival and tumor burden of the *Apc*^het^ *Usp7* cKO mice at later time points (Figures 4E, S4G, and S4H). We also observed colitis pathology in all *Apc*^het^ *Usp7* cKO mice (n = 11), while only 2 out of 13 *Apc*^het^ control animals showed minor degrees of inflammation (Figures 4F and 4G). In addition, enteritis was also observed in 2 out of 5 *Apc*^het^ *Usp7* cKO animals culled at around day 90 and was more abundant (4 out of 5 animals) at long time points (day 200) (Figures 4H and S4I). Similar to *Apc*^min^ mice, we confirmed the involvement of macrophages/monocytes in this inflammatory process as observed by CD68 staining (Figure S4J). To determine if gut inflammation mediated by USP7 deficiency in *Apc*^het^ mice is early or late onset, we collected intestinal tissues 15 and 30 days after tamoxifen induction for analysis. Histopathological analysis revealed that *Apc*^het^ *Usp7* cKO mice displayed signs of colitis and enteritis as early as day 15, indicating that the observed inflammation is an early event (Figure S4K). The data support the notion of inflammation-associated tumorigenesis in the *Apc*^het^ *Usp7* mice.

Since *Usp7* depletion induces gut inflammation in animals with germline *Apc* mutation only but not in normal WT mice, we asked if *Usp7* inhibition can improve the survival of sporadic intestinal tumor. We switched to an inducible sporadic mouse model using *Lgr5* EGFP-IRES^cre^ (Barker et al., 2007) to delete *Apc* and/or *Usp7* specifically in intestinal stem cells. *Lgr5* EGFP-IRES^cre^ *Apc*^fl/fl^ mice developed hyperproliferation and dysplasia, which were reduced upon *Usp7* inhibition (Figure S4L). Remarkably, we observed a significant increase in the lifespan of the mice upon *Usp7* deletion (Figure 4I), indicating that *Usp7* can be an effective target for sporadic CRC. Altogether, these results indicate that inhibition of *Usp7* suppresses intestinal tumor development and progression mediated by *Apc*-truncating mutations. Importantly, our data show that *Usp7* depletion is well tolerated in normal intestine but may have an adverse effect on *Apc* heterozygous (*Apc*^+/−^) intestinal cells due to haplo-deficiency.

Previous data from our lab (Novellasdemunt et al., 2017) showed that genetic *USP7* deletion translates a stronger phenotype than chemical inhibition. We asked if treatment of USP7 inhibitor to germline mutant *Apc*^min^ mice will be less efficacious in suppressing USP7 function than genetic deletion, hence offering a potential therapeutic window to suppress tumor growth without causing gut inflammation. To assess the hypothesis, we treated the *Apc*^min^ mice with either a USP7 inhibitor, P22077, or vehicle for 21 days. As expected, P22077-treated mice showed a significant decrease in tumor number and size compared with the vehicle control (Figures 4J–4L). Intriguingly, no colitis or enteritis was observed in any of the collected mice, suggesting that pharmacological inhibition of USP7 could be a safe option for patients with germline *APC* mutation. Further experimentation with a larger cohort of animals and longer drug treatments will be needed to validate the findings. Together, our results imply that USP7 can be an effective therapeutic target for both sporadic and germline *APC*-mutated CRCs.

### USP7 inhibition suppresses WNT signaling and growth of human CRC PDOs carrying *APC*-truncating mutations

**Figure 5:**
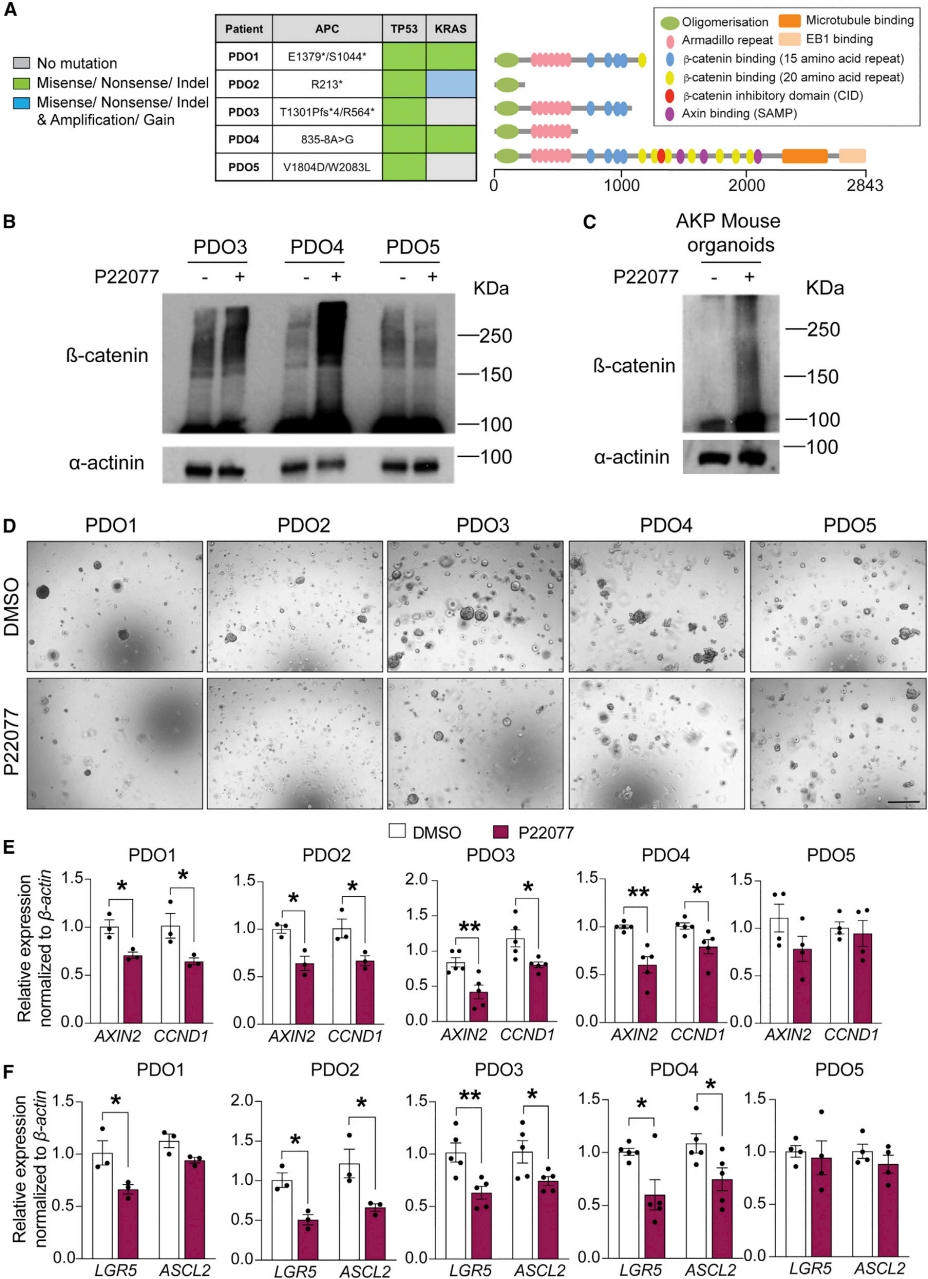
*USP7* inhibition suppresses WNT signaling and restores β-catenin ubiquitination in human PDOs carrying *APC* truncating mutations (A) Left: mutation status of the key driver genes in PDOs 1–5. An asterisk (^∗^) indicates truncated protein. Right: schematic representation of the APC protein products with different domains depicted. Numbers indicate codon numbers. (B and C) Human PDOs (B) and mouse organoids (C) were treated with P22077 or vehicle (DMSO) as indicated, and lysates were collected for immunoblotting of β-catenin and control α-actinin (n = 3 biological replicates per each PDO or AKP mouse organoid). (D) Representative images of colony-formation assay of organoids treated with P22077 or vehicle DMSO. Scale bar, 500 μm. (E and F) mRNA expression of the indicated genes was analyzed by qRT-PCR in the organoids from (D). Data are presented as fold change normalized to *β-actin* control (at least n = 4 biological replicates per each PDO donor). Error bars represent ± SE. p values were determined using the unpaired two-sided t test (^∗^p < 0.05; ^∗∗^p < 0.01).

To further validate the therapeutic potential in human CRC, we treated PDOs with the USP7 inhibitor P22077 that has been previously used for *in vivo* studies (Fan et al., 2013; Novellasdemunt et al., 2017). Human PDOs were established from 5 patients with CRC carrying different *APC* mutations (Figure 5A). PDOs 1–3 harbor different truncating *APC* mutations, all lacking the β-catenin inhibitory domain (CID) critical for pathological WNT activation and tumor transformation via USP7 binding (Novellasdemunt et al., 2017). PDO4 carries a splice variant mutation at the −8 position upstream of exon 8 of the *APC* gene, leading to early frameshift and premature truncation (Fostira et al., 2010). PDO5 has 2 missense mutations of *APC* after the CID domain toward the 3′ end that are predicted to yield full-length (FL), non-pathological APC protein. Microsatellite repeat analysis showed that PDO5 should be classified as an MSI CRC with WT APC and low WNT activity.

We have previously shown that treatment of different *APC*-mutated CRC cell lines with USP7 inhibitor P22077 downregulates WNT activity by restoring β-catenin ubiquitination (Novellasdemunt et al., 2017). To validate if the same mechanism of action applies in PDOs, we performed western blot analysis of β-catenin in PDOs 3 and 5 in the presence or absence of USP7 inhibitor. Indeed, an increase in β-catenin ubiquitination was observed upon USP7 inhibition in PDO3 and PDO4 carrying *APC*-truncating mutations, while no significant difference was observed in APC-FL PDO5 (Figure 5B). Consistently, P22077-treated mouse CRC organoids carrying *Apc*, *Kras*^*G12D*^, and *P53*^*null*^ (AKP) mutations also showed restoration of β-catenin ubiquitination (Figure 5C). We then tested the therapeutic potential of the USP7 inhibitor in human PDOs. Treatment of the PDOs with P22077 resulted in a growth reduction in the APC-truncated PDOs 1–4 but not the APC-FL PDO5 (Figures 5D and S5A). Consistently, organoid formation efficiency was also reduced in PDOs 1–4 (Figure S5B). Quantitative RT-PCR analysis confirmed that the WNT target genes (*AXIN2* and *CCND1*) and stem cell markers (*LGR5* and *ASCL2*) were significantly reduced in PDOs 1–4 upon USP7 inhibition, while PDO5 was unaffected by the treatment (Figures 5E and 5F). These results are in concordance with the *in vivo* mouse data that USP7 inhibition suppresses hyperproliferation and tumor development specifically in APC-deficient intestine.

Next, we explored if USP7 inhibition could be used as an adjuvant therapy to fluorouracil (5-FU), a common chemotherapy given to patients with CRC in the clinic. CellTiter-Glo viability analysis showed that the 5-FU-mediated growth suppression efficiency varied among the 5 PDOs, where PDOs 3 and 4 responded better than PDOs 1, 2, and 5 (Figures S5C and S5D). Combination treatment of 5-FU and P22077 showed additive growth suppression compared with 5-FU alone in PDOs 2–4 (Figure S5C). In particular, the organoid growth of PDOs 3 and 4 was nearly completely abolished in the combined treatment (Figures S5C and S5D). On the other hand, P22077 treatment did not further increase the 5-FU-mediated growth suppression in PDO5. Similar results were observed when analyzing organoid formation capacity (Figure S5E). These data suggest that USP7 inhibition may be used in combination with current chemotherapeutic drugs such as 5-FU in *APC*-mutated CRCs.

**Figure 6:**
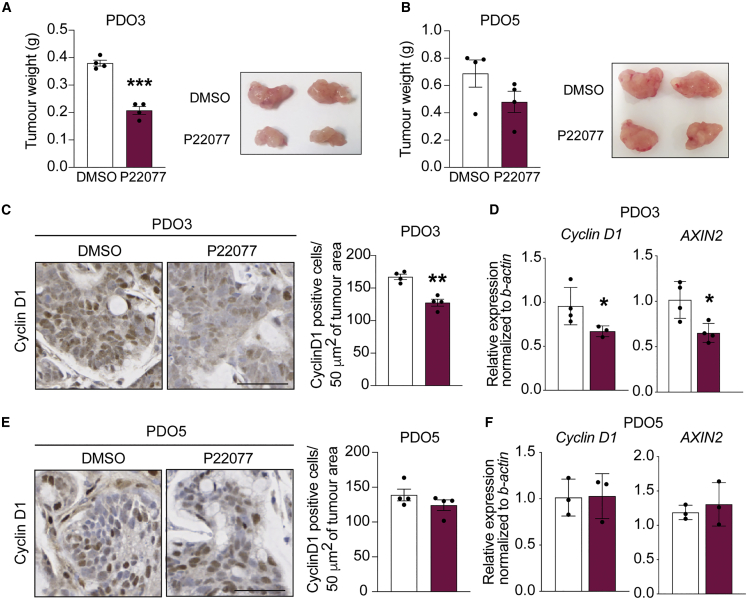
USP7 inhibition suppresses tumor growth *in vivo* in APC-truncated PDO-derived xenografts (A and B) Tumor weights of PDO3 (A) and PDO5 (B) between DMSO control and P22077 treatment groups (30 mg/kg) were measured at the end of treatment (20 days) (n = 4 biological replicates per condition). Representative images of tumors derived from PDOs 3 (A) and 5 (B) at the end of treatment. Error bars represent ± SE. p values were determined using the unpaired two-sided t test (^∗∗∗^p < 0.001). (C and E) Representative images of cyclin D1 stainings from DMSO- or P22077-treated PDO3- (C) or PDO5-derived (E) tumors. Scale bar, 50 μm. Left panel: quantitation of cyclin D1-positive cells shown on the right panel. Each dot represents the average of at least 6 different areas of 50 μm^2^ per tumor. Data are mean ± SE. n = 4 biological replicates per group. p values were determined using the unpaired two-sided t test (^∗∗^p < 0.01). (D and F) mRNA expression of the indicated genes was analyzed by qRT-PCR in tumors derived from PDOs 3 (D) and 5 (F). Data are presented as fold change normalized to *β-actin*. Error bars represent ± SE. At least n = 3 experimental replicates from three independent experiments. p values were determined using the unpaired two-sided t test (^∗^p < 0.05).

Finally, we tested the therapeutic potential of USP7 inhibitor *in vivo* by transplanting the PDOs subcutaneously into immunodeficient mice. To validate the *in vitro* treatment data, the good (PDO3) and poor (PDO5) responders of the USP7 inhibitor were selected for the xenograft experiment. USP7 inhibitor P22077 or vehicle control (DMSO) were administered daily by intraperitoneal injections (Novellasdemunt et al., 2017). Treatment by P22077 of the PDO3 xenografts significantly suppressed tumor growth compared with vehicle treatment (Figure 6A). In contrast, PDO5 xenografts did not show a significant effect on tumor development upon P22077 treatment (Figure 6B). Similar to our previous observation (Novellasdemunt et al., 2017), treatment of the USP7 inhibitor *in vivo* did not show any detectable health problems or weight loss (Figure S6A). In addition, histological analysis of the P22077-treated PDO3 tumors showed a decreased expression of the WNT target gene CCND1 (Figure 6B). Expression analysis of PDO3 further confirmed the downregulation of *CCND1* and *AXIN2* upon P22077 treatment (Figures 6C and 6D). On the other hand, expression of *CCND1* and *AXIN2* was unchanged in the P22077-treated PDO5 tumors (Figures 6E and 6F). Of note, cleaved caspase3 expression was unaltered in both PDO xenografts upon treatment, suggesting that P22077-mediated tumor suppression is not caused by increased apoptosis (Figure S6B). We further validated the findings in another good responder PDO4. Although PDO4 displayed higher heterogeneity in tumor size (Figure S6C), P22077-treated organoids showed consistent reduction of tumor growth over the course of treatment (Figure S6E). Altogether, the data support the notion that USP7 inhibition can be used for treatment of *APC*-mutated CRC.

## Discussion

The major genetic events that cause CRC have been extensively characterized over the past decades. In particular, loss-of-function mutations of *APC* represent the key driver mutation for over 80% of CRCs. It has been previously shown that CRCs carrying *KRAS* and *P53* mutations remain strictly dependent on APC loss (Dow et al., 2015), indicating that the WNT signaling pathway is an important therapeutic target for treatment of CRC. However, despite decades of research, no approved drugs targeting WNT signaling in *APC*-mutated cancer exist. One of the major challenges is the crucial role of the WNT pathway in adult tissue homeostasis, making it difficult to develop safe and effective WNT inhibitors for treating CRC without on-target toxicity (Clevers and Nusse 2012; Novellasdemunt et al., 2015). There is an urgent unmet need to develop a new generation of WNT inhibitors that exhibit tumor specificity.

We have recently identified USP7 as a tumor-specific target in CRC carrying APC-truncating mutations (Novellasdemunt et al., 2017). We showed that APC truncations lacking the CID make β-catenin vulnerable to USP7 deubiquitination: depletion of USP7 in *APC*-mutant CRC cell lines and mouse organoids inhibits WNT and suppresses cell growth. Importantly, we demonstrated that the USP7 is dispensable for WNT activity and cell viability in APC-WT cells, highlighting its therapeutic potential for CRC treatment.

Contrasting with our findings, it has been recently reported that USP7 functions as a potent negative WNT regulator by deubiquitinating AXIN in normal HEK293T cells, mesenchymal stem cells C3H10T1/2, and bone-marrow-derived stroma cells ST2 (Ji et al., 2019). This raises uncertainty about the roles of USP7 in WNT regulation and the safe use of USP7 inhibitors for treating CRC. To address these concerns, we have generated a number of transgenic mouse strains to test the functional role of *Usp7* in adult intestinal homeostasis and tumorigenesis *in vivo*. Both short- and long-term intestine-specific loss of *Usp7* reveals no major phenotype in WT animals, supporting the notion that *Usp7* is dispensable for WNT signaling and tissue homeostasis in the normal WT intestine. In contrast, *Usp7* depletion in *Apc*^fl/fl^ mice ameliorates the acute crypt proliferation and survival, with reduced expression of WNT target genes and stem cell markers accompanied by increased differentiation. The data are consistent with a previous finding whereby restoring APC function promotes tissue differentiation and tumor regression (Dow et al., 2015).

We further investigated the role of *Usp7* in CRC using two independent mouse intestinal tumor models (*Apc*^min^ and *Apc*^het^) that mimic human patients with FAP with germline *APC* mutations predisposed to CRC development. Loss of *Usp7* significantly reduces tumor numbers and tumor grade in both models, indicating that *Apc*-deficient tumor development and progression is *Usp7* dependent. This is further validated in human PDOs where treatment of USP7 inhibitor suppresses the growth of APC-truncated, but not APC-FL, CRCs, supporting the idea of using USP7 inhibitors for CRC treatment. Nevertheless, the survival of both mouse *Apc*^+/−^ tumor models was not improved despite the complete absence of tumor development in *Apc*^het^ *Usp7* cKO animals 90 days after induction. To our surprise, we observed intestinal tumor development at later time points in both *Apc*^min^ *Usp7* cKO and *Apc*^het^ *Usp7* cKO animals. Histological analysis showed colitis and enteritis inflammation in both *Apc*^min^ *Usp7* cKO and *Apc*^het^ *Usp7* cKO mice at approximately 3–4 months of age, which was not detected in the WT *Usp7* cKO animals even after 1.5 years of deletion. We further showed that inflammation happened as early as 15 days after induction in *Apc*^het^ *Usp7* cKO animals. The results suggest that *Usp7* deletion is well tolerated in *Apc* WT normal cells but may cause toxicity in *Apc* heterozygous cells. Conceivably, heterozygous loss of APC may partially expose β-catenin to USP7, resulting in a moderate dependency on USP7 in those cells. On the other hand, *Usp7* depletion in *Lgr5* EGFP-IRES^cre^ *Apc*^fl/fl^ tumor model showed clear survival advantage, indicating that USP7 is an effective target for sporadic CRC. Interestingly, our data showed that pharmacological inhibition of USP7 *in vivo* in *Apc*^min^ mice was well tolerated and resulted in a significant decrease in tumor numbers with no signs of colitis or enteritis after 21 days treatment. This is probably due to the lower efficiency of targeting USP7 function pharmacologically than genetic deletion. Our findings imply that a USP7 inhibitor can be safe and effective in treating sporadic *APC*-mutated CRCs lacking the CID domain (Novellasdemunt et al., 2017) as well as patients with FAP with germline *APC* mutations. Further studies will be needed to understand the colitis development in the *Apc*^min^/*Apc*^het^ *Usp7* cKO mice.

USP7 has been previously shown to regulate p53-dependent apoptosis by controlling the levels of P53 and MDM2 (Li et al., 2002; Meulmeester et al., 2005). Our previous study showed that USP7-mediated WNT activation is P53 independent (Novellasdemunt et al., 2017). Indeed, we did not observe any transactivation of P21 protein expression in the *Usp7* cKO intestine. Importantly, P53 is mutated in all human PDOs used in this study regardless of their response to the USP7 inhibitor treatment, which is consistent with our previous observation that the WNT-regulatory role of USP7 is P53 independent. Our PDO treatment data further suggest that USP7 inhibition can be used either alone or in combination with chemotherapeutic agents already being utilized clinically in treating CRC. It will be interesting to explore if P53 plays a role in the combined treatment of chemotherapy and USP7 inhibitor.

It is also worth noting that genetic deletion of *Usp7 in vivo* displays a stronger effect of tumor suppression than the treatment of the USP7 inhibitor in PDOs. One likely explanation is the efficacy of the USP7 inhibitor P22077. Although several second-generation USP7 inhibitors have been described with increased selectivity and potency (Kategaya et al., 2017; Pozhidaeva et al., 2017; Turnbull et al., 2017; Gavory et al., 2018), there is limited data on the use of these inhibitors *in vivo*. Given that USP7 regulates both P53 and β-catenin, and potentially other substrates, it will be worth exploring if any of these (and other) new USP7 inhibitors target specifically the interaction between USP7 and β-catenin to increase drug selectivity and efficacy.

In conclusion, our data provide robust *in vivo* evidence of USP7 as a tumor-specific therapeutic target in *APC*-mutated CRCs. Our finding is inconsistent with the previous suggestion of USP7 as a potent negative WNT regulator (Ji et al., 2019), at least not in the intestine. However, it should be noted that the current study focuses mainly on the intestinal tract. We cannot exclude the possibility that USP7 may play a different role in other systems such as osteoblast and adipocyte differentiation, as previously suggested (Ji et al., 2019). Further investigation will be needed to fully characterize the functional roles of USP7 in different tissue systems.

## Experimental procedures

### Resource availability

#### Corresponding author

Requests for resources, reagents and protocols should be addressed to and will be fulfilled by the corresponding author, Vivian S.W. Li (vivian.li@crick.ac.uk).

#### Materials availability

This study did not generate new unique reagents.

### Antibodies and other reagents

β-Catenin (610154, BD); caspase-3 (9661L, Cell Signaling); cyclin D1 (2978S, Cell Signaling); lysozyme (A0099, Dako); Sox9 (AB5335, Millipore); Olfm4 (39141S, Cell Signaling); USP7 (Bethyl Laboratories); CD44 (MAB2137, Merck); and keratin 20 (13063S, Cell Signaling) were used in immunohistochemistry analysis. α-Actinin (sc-15335) and β-catenin (610154, BD) were used for western blot analysis. DUB inhibitor VI P22077 (Calbiochem) was resuspended in DMSO at 10 mM (for organoid experiments) or 15 mg/mL (for mice experiments).

### Animal procedures

All animal regulated procedures were carried out according to Project License constraints (PEF3478B3 and 70/8560) and home office guidelines and regulations. In accordance with replacement, reduction, and refinement (the 3Rs), the smallest sample size was chosen that could show a significant difference. Usp7^fl/fl^ mice were obtained from Kon et al. (2011). Animals of both sexes at age 6–7 weeks on C57/BL6J background were used for the different experimental conditions and harvested as indicated.

Tamoxifen was injected intraperitoneally for 3 consecutive days (1.5 mg/10 g of mouse weight) from a 20 mg/mL stock solution. For experiments involving APC^min^ mice, animals were injected once a week with the same dose of tamoxifen after the first week of injections to ensure proper Usp7 deletion. For experiments with Villin^CreERT2^; Usp7^fl/fl^ mice that were allowed to age up to 1.5 years, one injection of the same dose of tamoxifen was injected once a month to ensure proper Usp7 deletion. For proliferation analysis, EdU (Life Technologies) was injected intraperitoneally (0.3 mg/10 g of mouse weight) from a 10 mg/mL stock solution, and mice were culled 2 h after the injection. P22077 (30 mg/kg) was injected intraperitoneally daily for 21 days into Apc^min^ mice.

### Quantification and statistical analysis

Statistical analyses were performed using GraphPad Prism 8 software. Statistical details and sample numbers are specified in the figure legends. For parametric data, statistical significance was determined using Student’s unpaired, two-tailed t test. For survival experiments, log rank (Mantel-Cox) test was used. p values are represented as ^∗^p < 0.05; ^∗∗^p < 0.01; ^∗∗∗^p < 0.001.

## Author contributions

L.N. designed the study, conducted the experiments, analyzed the data, and wrote the manuscript. A.K., A.B., and C.H. performed and analyzed the mouse experiments. A.S.-B. analyzed mouse tissues. G.V., D.R., F.C., and N.V. harvested human CRC tissues and generated PDOs. A.R. provided technical support to PDO characterization. V.S.W.L. designed the study and wrote the manuscript.

## Data Availability

This study did not generate datasets.

## Supplemental information

Document S1. Figures S1–S6 and supplemental experimental procedures

Document S2. Article plus supplemental information

## References

[bib1] Aberle H., Bauer A., Stappert J., Kispert A., Kemler R. (1997). beta-catenin is a target for the ubiquitin-proteasome pathway. EMBO J..

[bib2] Andrew H.K., Chiorean E.G., Kwak E.L., Lenz H.-J., Nadler P.I., Wood D.L., Fujimori M., Inada T., Kouji H., McWilliams R.R. (2016). Final results of a phase Ib dose-escalation study of PRI-724, a CBP/beta-catenin modulator, plus gemcitabine (GEM) in patients with advanced pancreatic adenocarcinoma (APC) as second-line therapy after FOLFIRINOX or FOLFOX. J. Clin. Oncol..

[bib3] Barker N., van Es J.H., Kuipers J., Kujala P., van den Born M., Cozijnsen M., Haegebarth A., Korving J., Begthel H., Peters P.J., Clevers H. (2007). Identification of stem cells in small intestine and colon by marker gene Lgr5. Nature.

[bib4] Bray F., Ferlay J., Soerjomataram I., Siegel R.L., Torre L.A., Jemal A. (2018). Global cancer statistics 2018: GLOBOCAN estimates of incidence and mortality worldwide for 36 cancers in 185 countries. CA. Cancer J. Clin..

[bib5] Cancer Genome Atlas Network (2012). Comprehensive molecular characterization of human colon and rectal cancer. Nature.

[bib6] Chen B., Dodge M.E., Tang W., Lu J., Ma Z., Fan C.W., Wei S., Hao W., Kilgore J., Williams N.S. (2009). Small molecule-mediated disruption of Wnt-dependent signaling in tissue regeneration and cancer. Nat. Chem. Biol..

[bib7] Clevers H., Nusse R. (2012). Wnt/beta-catenin signaling and disease. Cell.

[bib8] DeAlmeida V.I., Miao L., Ernst J.A., Koeppen H., Polakis P., Rubinfeld B. (2007). The soluble wnt receptor Frizzled8CRD-hFc inhibits the growth of teratocarcinomas in vivo. Cancer Res..

[bib9] Dow L.E., O'Rourke K.P., Simon J., Tschaharganeh D.F., van Es J.H., Clevers H., Lowe S.W. (2015). Apc restoration promotes cellular differentiation and reestablishes crypt homeostasis in colorectal cancer. Cell.

[bib10] Fan Y.H., Cheng J., Vasudevan S.A., Dou J., Zhang H., Patel R.H., Ma I.T., Rojas Y., Zhao Y., Yu Y. (2013). USP7 inhibitor P22077 inhibits neuroblastoma growth via inducing p53-mediated apoptosis. Cell Death Dis..

[bib11] Fostira F., Thodi G., Sandaltzopoulos R., Fountzilas G., Yannoukakos D. (2010). Mutational spectrum of APC and genotype-phenotype correlations in Greek FAP patients. BMC Cancer.

[bib12] Gaspar C., Fodde R. (2004). APC dosage effects in tumorigenesis and stem cell differentiation. Int. J. Dev. Biol..

[bib13] Gavory G., O'Dowd C.R., Helm M.D., Flasz J., Arkoudis E., Dossang A., Hughes C., Cassidy E., McClelland K., Odrzywol E. (2018). Discovery and characterization of highly potent and selective allosteric USP7 inhibitors. Nat. Chem. Biol..

[bib14] Grivennikov S.I. (2013). Inflammation and colorectal cancer: colitis-associated neoplasia. Semin. Immunopathol..

[bib15] Groden J., Thliveris A., Samowitz W., Carlson M., Gelbert L., Albertsen H., Joslyn G., Stevens J., Spirio L., Robertson M. (1991). Identification and characterization of the familial adenomatous polyposis coli gene. Cell.

[bib16] Gurney A., Axelrod F., Bond C.J., Cain J., Chartier C., Donigan L., Fischer M., Chaudhari A., Ji M., Kapoun A.M. (2012). Wnt pathway inhibition via the targeting of Frizzled receptors results in decreased growth and tumorigenicity of human tumors. Proc. Natl. Acad. Sci. USA.

[bib17] Huang S.M.A., Mishina Y.M., Liu S., Cheung A., Stegmeier F., Michaud G.A., Charlat O., Wiellette E., Zhang Y., Wiessner S. (2009). Tankyrase inhibition stabilizes axin and antagonizes Wnt signalling. Nature.

[bib18] Ji L., Lu B., Zamponi R., Charlat O., Aversa R., Yang Z., Sigoillot F., Zhu X., Hu T., Reece-Hoyes J.S. (2019). USP7 inhibits Wnt/beta-catenin signaling through promoting stabilization of Axin. Nat. Commun..

[bib19] Jimeno A., Gordon M., Chugh R., Messersmith W., Mendelson D., Dupont J., Stagg R., Kapoun A.M., Xu L., Uttamsingh S. (2017). A first-in-human phase I study of the anticancer stem cell agent ipafricept (OMP-54F28), a decoy receptor for wnt ligands, in patients with advanced solid tumors. Clin. Cancer Res..

[bib20] Joslyn G., Carlson M., Thliveris A., Albertsen H., Gelbert L., Samowitz W., Groden J., Stevens J., Spirio L., Robertson M. (1991). Identification of deletion mutations and three new genes at the familial polyposis locus. Cell.

[bib21] Jung Y.S., Park J.I. (2020). Wnt signaling in cancer: therapeutic targeting of Wnt signaling beyond beta-catenin and the destruction complex. Exp. Mol. Med..

[bib22] Kategaya L., Di Lello P., Rougé L., Pastor R., Clark K.R., Drummond J., Kleinheinz T., Lin E., Upton J.P., Prakash S. (2017). USP7 small-molecule inhibitors interfere with ubiquitin binding. Nature.

[bib23] Kinzler K.W., Nilbert M.C., Su L.K., Vogelstein B., Bryan T.M., Levy D.B., Smith K.J., Preisinger A.C., Hedge P., McKechnie D. (1991). Identification of FAP locus genes from chromosome 5q21. Science.

[bib24] Kitagawa M., Hatakeyama S., Shirane M., Matsumoto M., Ishida N., Hattori K., Nakamichi I., Kikuchi A., Nakayama K., Nakayama K. (1999). An F-box protein, FWD1, mediates ubiquitin-dependent proteolysis of beta-catenin. EMBO J..

[bib25] Kon N., Zhong J., Kobayashi Y., Li M., Szabolcs M., Ludwig T., Canoll P.D., Gu W. (2011). Roles of HAUSP-mediated p53 regulation in central nervous system development. Cell Death Differ..

[bib26] Lau T., Chan E., Callow M., Waaler J., Boggs J., Blake R.A., Magnuson S., Sambrone A., Schutten M., Firestein R. (2013). A novel tankyrase small-molecule inhibitor suppresses APC mutation-driven colorectal tumor growth. Cancer Res..

[bib27] Li M., Chen D., Shiloh A., Luo J., Nikolaev A.Y., Qin J., Gu W. (2002). Deubiquitination of p53 by HAUSP is an important pathway for p53 stabilization. Nature.

[bib28] Li V.S.W., Ng S.S., Boersema P.J., Low T.Y., Karthaus W.R., Gerlach J.P., Mohammed S., Heck A.J.R., Maurice M.M., Mahmoudi T., Clevers H. (2012). Wnt signaling through inhibition of beta-catenin degradation in an intact Axin1 complex. Cell.

[bib29] Liu C., Li Y., Semenov M., Han C., Baeg G.H., Tan Y., Zhang Z., Lin X., He X. (2002). Control of beta-catenin phosphorylation/degradation by a dual-kinase mechanism. Cell.

[bib30] Mariotti L., Pollock K., Guettler S. (2017). Regulation of Wnt/beta-catenin signalling by tankyrase-dependent poly(ADP-ribosyl)ation and scaffolding. Br. J. Pharmacol..

[bib31] Meulmeester E., Maurice M.M., Boutell C., Teunisse A.F.A.S., Ovaa H., Abraham T.E., Dirks R.W., Jochemsen A.G. (2005). Loss of HAUSP-mediated deubiquitination contributes to DNA damage-induced destabilization of Hdmx and Hdm2. Mol. Cell.

[bib32] Moore K.N., Gunderson C.C., Sabbatini P., McMeekin D.S., Mantia-Smaldone G., Burger R.A., Morgan M.A., Kapoun A.M., Brachmann R.K., Stagg R. (2019). A phase 1b dose escalation study of ipafricept (OMP54F28) in combination with paclitaxel and carboplatin in patients with recurrent platinum-sensitive ovarian cancer. Gynecol. Oncol..

[bib33] Nagase H., Nakamura Y. (1993). Mutations of the APC (adenomatous polyposis coli) gene. Hum. Mutat..

[bib34] Nishisho I., Nakamura Y., Miyoshi Y., Miki Y., Ando H., Horii A., Koyama K., Utsunomiya J., Baba S., Hedge P. (1991). Mutations of chromosome 5q21 genes in FAP and colorectal cancer patients. Science.

[bib35] Novellasdemunt L., Antas P., Li V.S.W. (2015). Targeting wnt signaling in colorectal cancer. A review in the theme: cell signaling: proteins, pathways and mechanisms. Am. J. Physiol. Cell Physiol..

[bib36] Novellasdemunt L., Foglizzo V., Cuadrado L., Antas P., Kucharska A., Encheva V., Snijders A.P., Li V.S.W. (2017). USP7 is a tumor-specific WNT activator for APC-mutated colorectal cancer by mediating beta-catenin deubiquitination. Cell Rep..

[bib37] Polakis P. (1995). Mutations in the APC gene and their implications for protein structure and function. Curr. Opin. Genet. Dev..

[bib38] Pozhidaeva A., Valles G., Wang F., Wu J., Sterner D.E., Nguyen P., Weinstock J., Kumar K.G.S., Kanyo J., Wright D., Bezsonova I. (2017). USP7-Specific inhibitors target and modify the enzyme's active site via distinct chemical mechanisms. Cell Chem. Biol..

[bib39] Sansom O.J., Reed K.R., Hayes A.J., Ireland H., Brinkmann H., Newton I.P., Batlle E., Simon-Assmann P., Clevers H., Nathke I.S. (2004). Loss of Apc in vivo immediately perturbs Wnt signaling, differentiation, and migration. Genes Dev..

[bib40] Shibata H., Toyama K., Shioya H., Ito M., Hirota M., Hasegawa S., Matsumoto H., Takano H., Akiyama T., Toyoshima K. (1997). Rapid colorectal adenoma formation initiated by conditional targeting of the Apc gene. Science.

[bib41] Su L.K., Kinzler K.W., Vogelstein B., Preisinger A.C., Moser A.R., Luongo C., Gould K.A., Dove W.F. (1992). Multiple intestinal neoplasia caused by a mutation in the murine homolog of the APC gene. Science.

[bib42] Turnbull A.P., Ioannidis S., Krajewski W.W., Pinto-Fernandez A., Heride C., Martin A.C.L., Tonkin L.M., Townsend E.C., Buker S.M., Lancia D.R. (2017). Molecular basis of USP7 inhibition by selective small-molecule inhibitors. Nature.

[bib43] Vogelstein B., Kinzler K.W. (2004). Cancer genes and the pathways they control. Nat. Med..

[bib44] Waaler J., Machon O., Tumova L., Dinh H., Korinek V., Wilson S.R., Paulsen J.E., Pedersen N.M., Eide T.J., Machonova O. (2012). A novel tankyrase inhibitor decreases canonical Wnt signaling in colon carcinoma cells and reduces tumor growth in conditional APC mutant mice. Cancer Res..

[bib45] Waaler J., Machon O., von Kries J.P., Wilson S.R., Lundenes E., Wedlich D., Gradl D., Paulsen J.E., Machonova O., Dembinski J.L. (2011). Novel synthetic antagonists of canonical Wnt signaling inhibit colorectal cancer cell growth. Cancer Res..

