# Supplementary material for: USP7 inactivation suppresses APC-mutant intestinal hyperproliferation and tumor development
Source: Stem Cell Reports. 2023 Jan 19;18(2):570–84. doi: 10.1016/j.stemcr.2022.12.013 (PMC9968985; doi:10.1016/j.stemcr.2022.12.013)
Supplement: Document S1. Figures S1–S6 and supplemental experimental procedures [file mmc1.pdf]

Stem Cell Reports, Volume *18*

Supplemental Information

USP7 inactivation suppresses APC-mutant intestinal hyperprolifera-tion and tumor development

Laura Novellasdemunt, Anna Kucharska, Anna Baulies, Colin Hutton, Georgios Vlachogiannis, Dimitra Repana, Andrew Rowan, A. Suárez-Bonnet, Francesca Ciccarelli, Nicola Valeri, and Vivian S.W. Li

Supplemental Information

USP7 inactivation suppresses APC-mutant intestinal hyperproliferation and tumor development

Laura Novellasdemunt[1,2], Anna Kucharska[1], Anna Baulies[1], Colin Hutton[1], Georgios Vlachogiannis[3], Dimitra Repana[4], Andrew Rowan[5], Suárez-Bonnet A[6], Francesca Ciccarelli[4], Nicola Valeri[3], Vivian S. W. Li[1, *]

Supplementary Figure 1

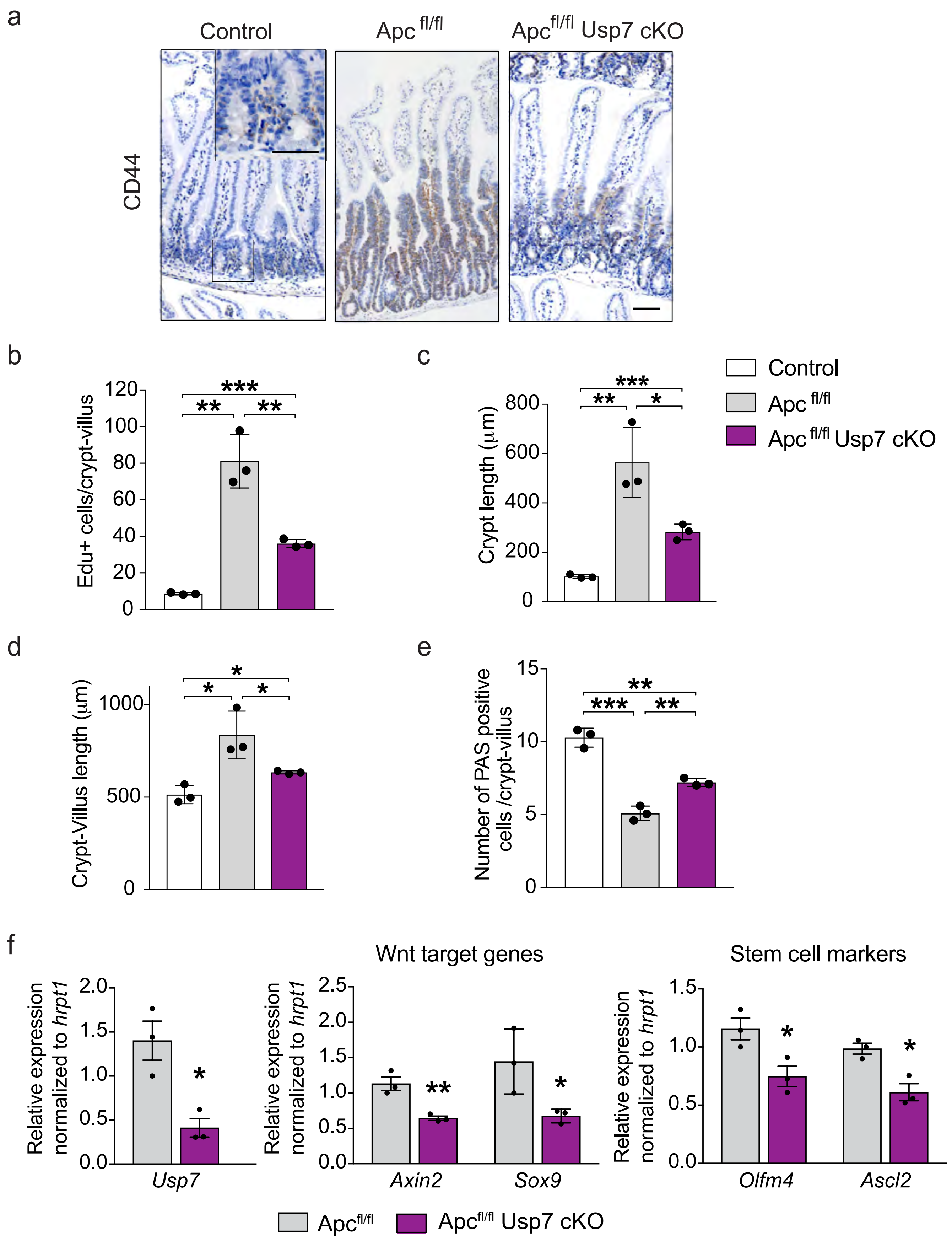

Supplementary Figure 2

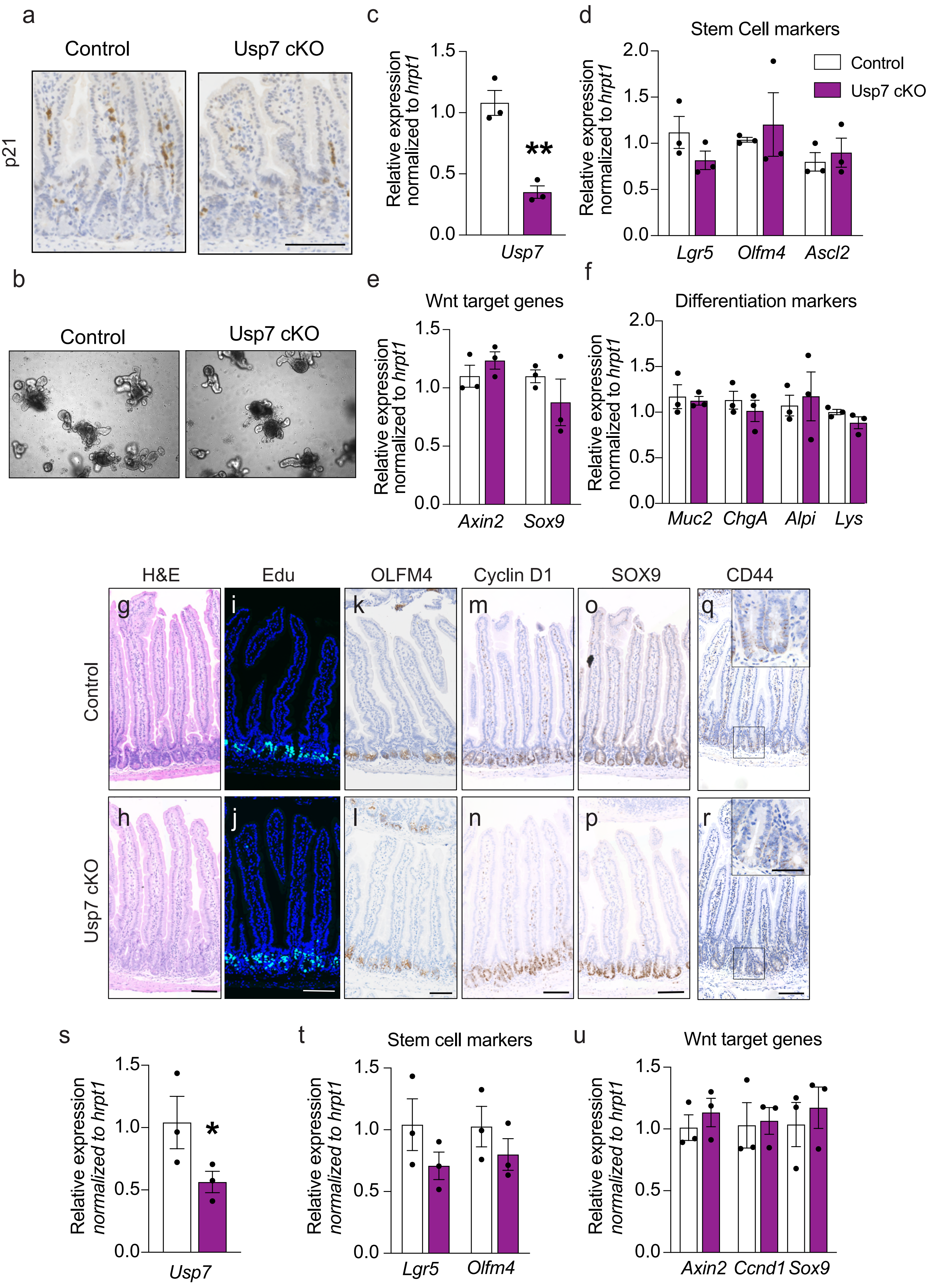

Supplementary Figure 3

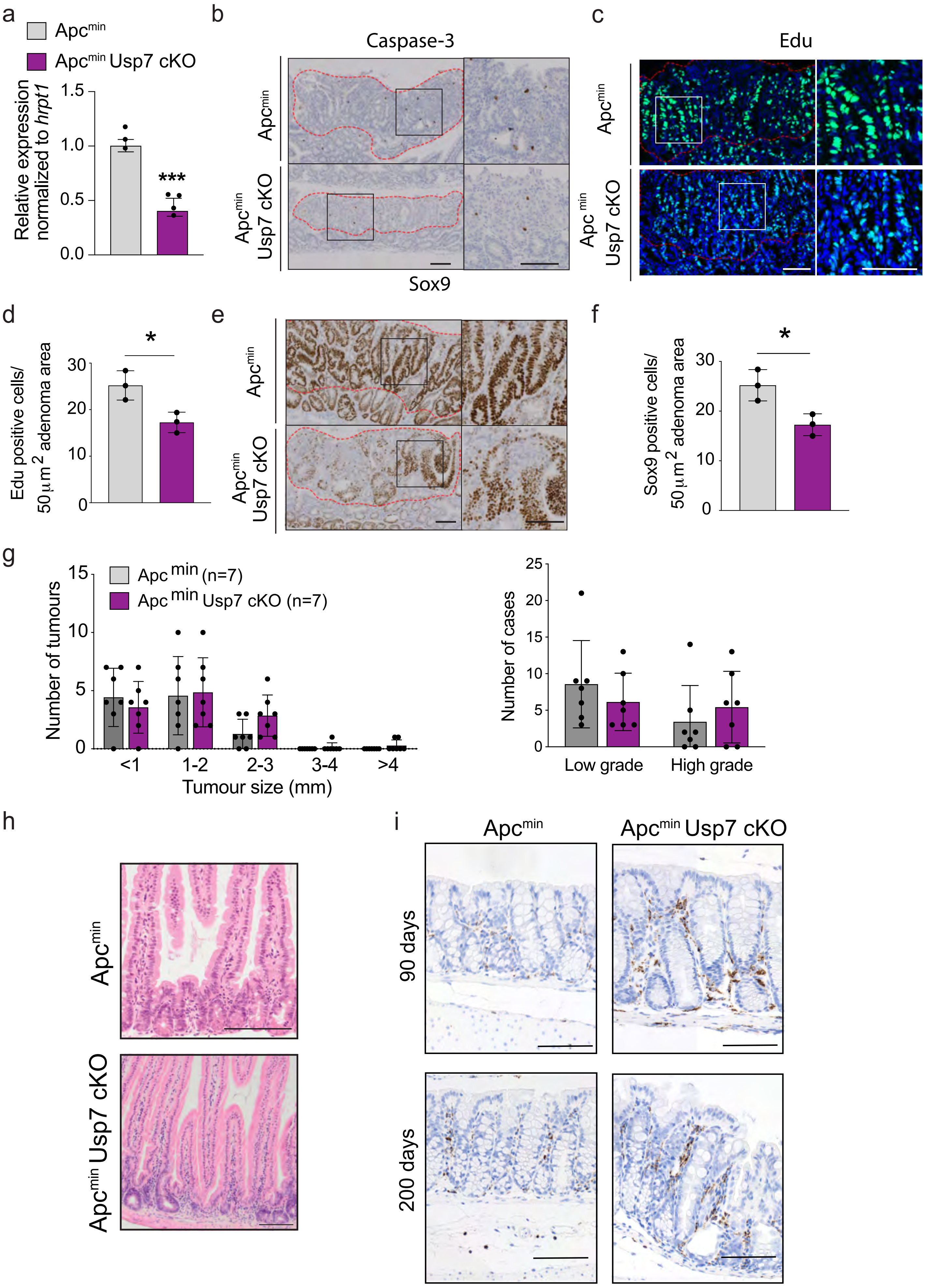

Supplementary Figure 4

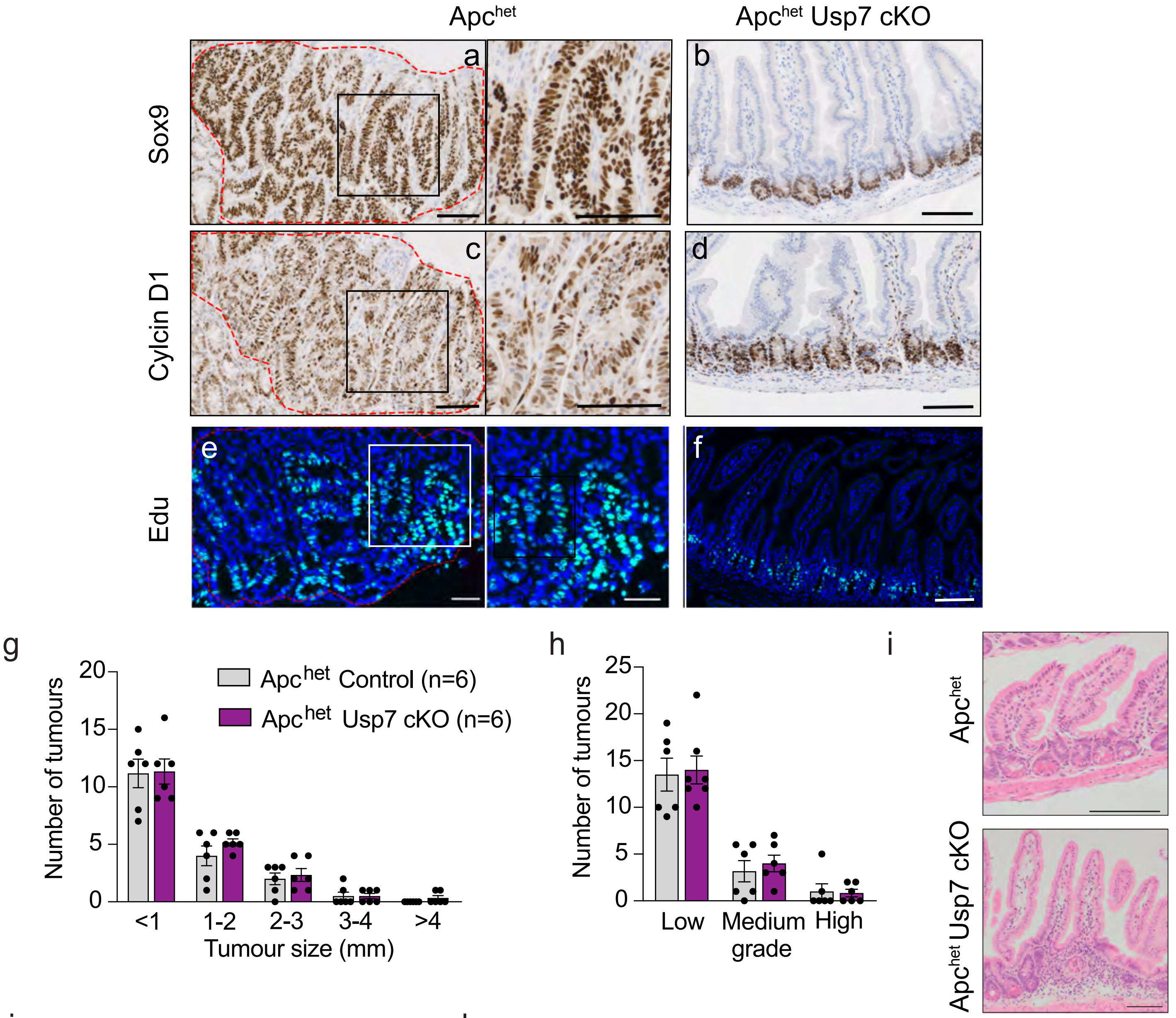

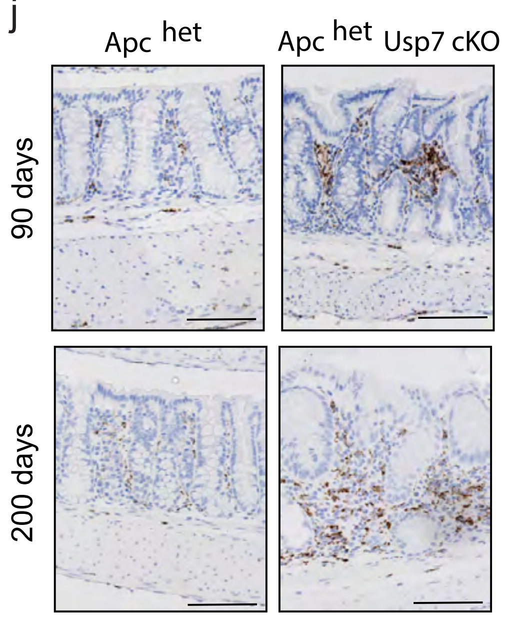

k

Enteritis/ Colitis score	Description	Day 15		Day 30	
		Apchet (n=4)	Apchet Usp7 cKO (n=6)	Apchet (n=8)	Apchet Usp7 cKO (n=6)
0	No enteritis/colitis	4/4	0/0	8/4	0/0
1	Minimal enteritis/colitis	0/0	0/0	0/3	0/0
2	Some enteritis/colitis present. Degree of inflammation	0/0	4/0	0/0	3/0
3	Minor enteritis/colitis	0/0	1/0	0/0	2/0
4	Mild enteritis/colitis	0/0	0/0	0/1	0/0
7	Crypt abscesses	0/0	1/6	0/0	1/6

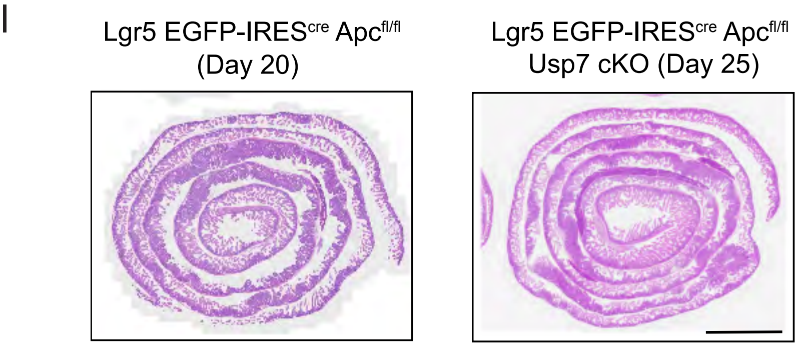

Supplementary Figure 5

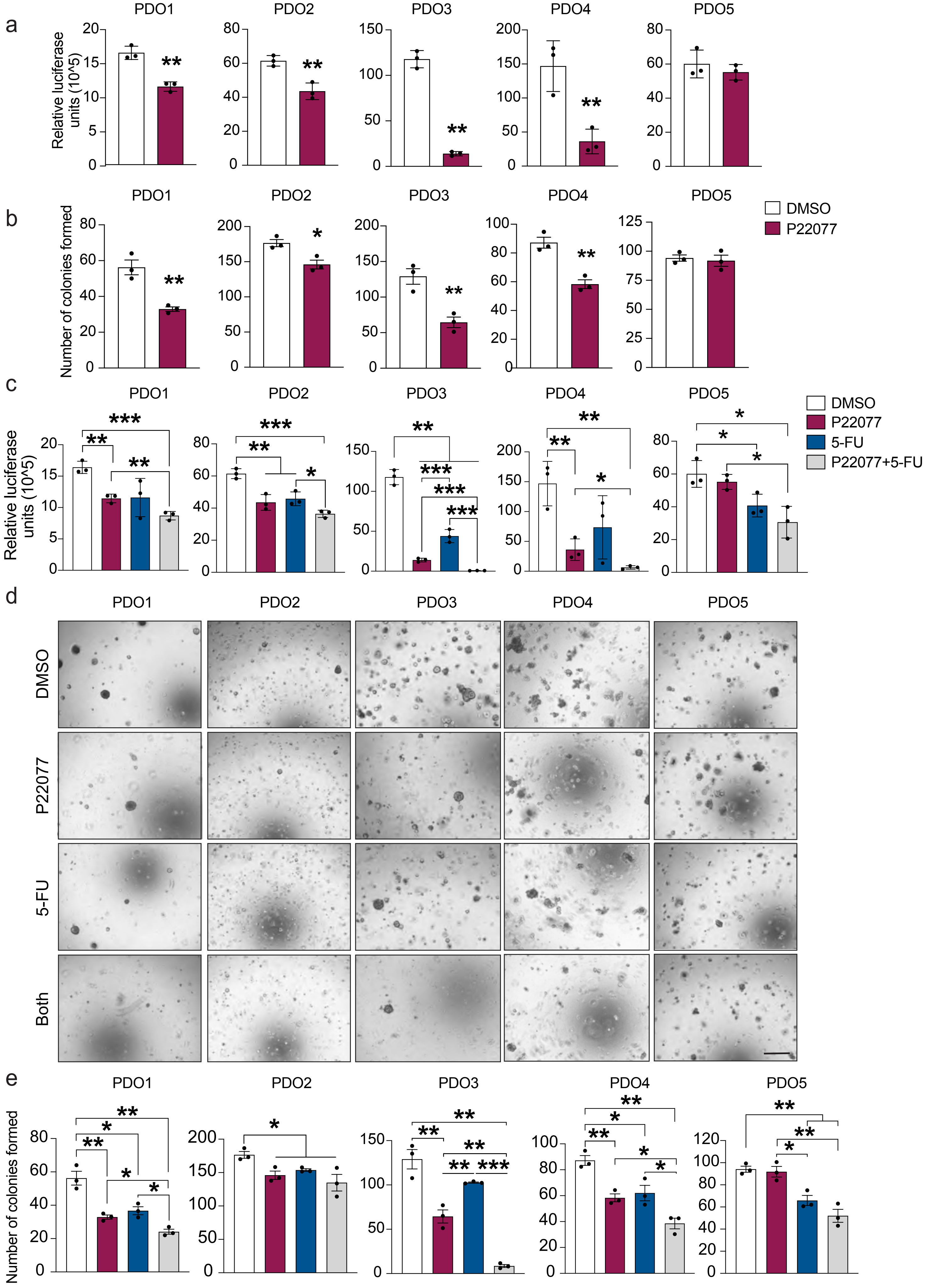

Supplementary Figure 6

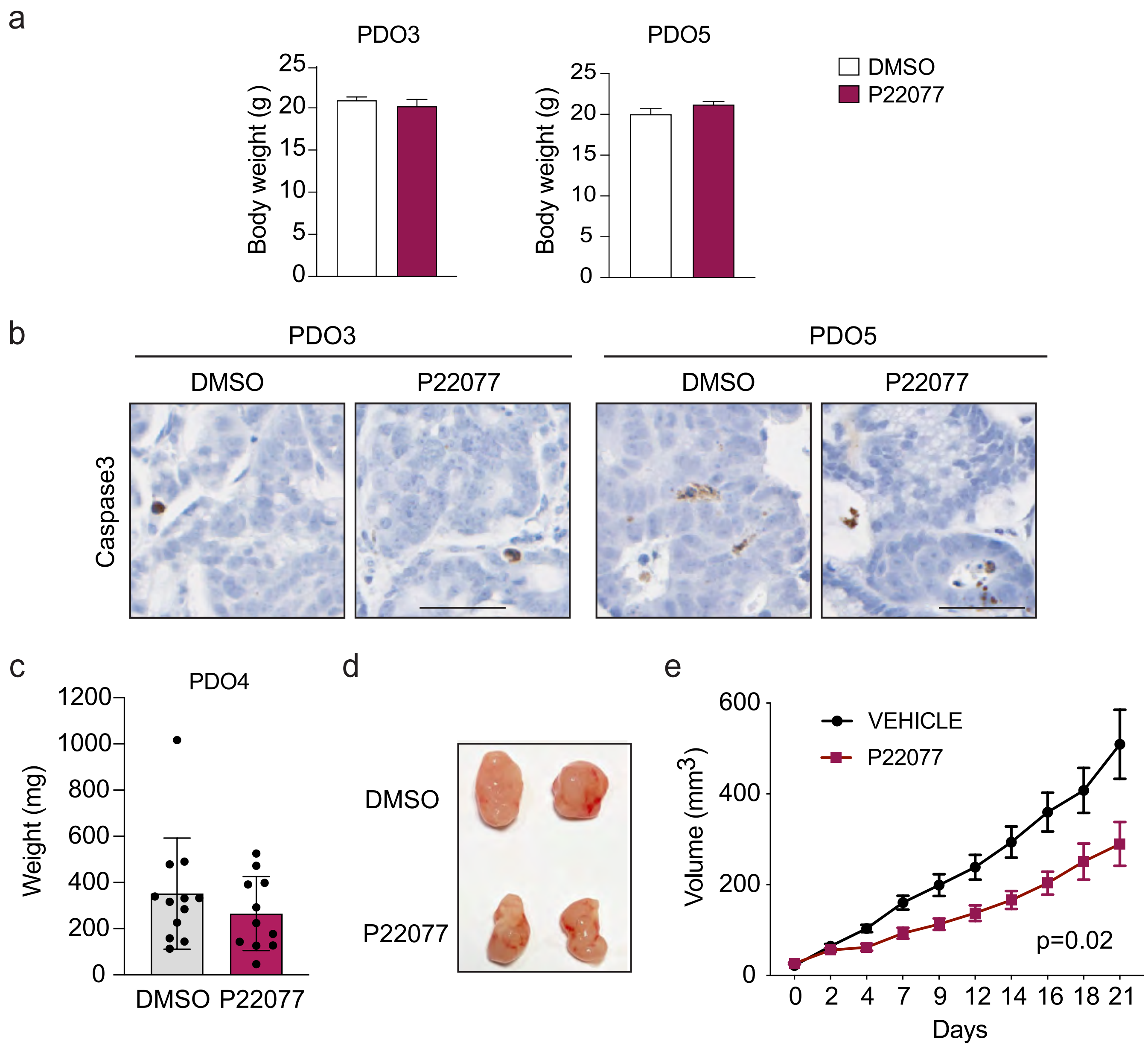

Supplemental Figure Legends

Figure S1. *Usp7* inhibition decreases WNT signaling and proliferation in organoids derived from *Apc*$^{fl/fl}$ mice, related to Figure 1. (**a**) Representative images of CD44 staining in *Villin*^{CreERT2} (Control), *Villin*^{CreERT2};*Apc*$^{fl/fl}$ (*Apc*$^{fl/fl}$) and *Villin*^{CreERT2};*Apc*$^{fl/fl}$;*Usp7*$^{fl/fl}$ (*Apc*$^{fl/fl}$ *Usp7*$^{fl/fl}$) at 6 dpi. Scale bar, 100 μm. (**b**) Quantitation of Edu positive cells from (Fig.1). Each dot represents the average of at least 10 crypts per animal. (**c**-**d**) Quantitation of crypt length (μm) (c) and crypt-villus length (μm) (d) from (Fig.1). Each dot represents the average of at least 10 crypts per animal. Data are mean ± standard error. n=3 biological replicates per group. (**e**) Quantitation of PAS staining (indicative of Goblet cells) positive cells from (Fig.1). Each dot represents the average of at least 20 crypts per animal. (**f**) mRNA expression of the indicated genes was analyzed by qRT-PCR in small intestinal organoids from (Fig. 1v). Data are presented as fold change normalized to *Hrpt1* (n=3 experimental replicates per condition). Error bars represent ± standard error. n=3 biological replicates per group. P-values were determined using the unpaired two-sided t-test (*P<0.05; **P<0.01; ***P<0.001).

Figure S2. Loss of *Usp7* in wild-type *Apc* mice maintains intestinal homeostasis, related to Figure 2. (a) Representative images of p21 staining in *Villin*^{CreERT2} (Control) and *Villin*^{CreERT2}; *Usp7*$^{fl/fl}$ (*Usp7* cKO) intestine. Scale bar, 100 μm. **(b)** Representative images of organoids derived from control and *Usp7* cKO mice at 7 dpi. Scale bar, 100 μm. (**c-f**) mRNA expression of the indicated genes was analyzed by qRT-PCR from organoids isolated from Control and *Usp7* cKO mice. Data are presented as fold change normalized to *β-actin* control in triplicate (n=3 mice as organoid donors per genotype). (**g**-**r**) Representative images of H&E staining (g-h), Edu (i-j), OLFM4 (k-l), Cyclin D1 (m-n), SOX9 (o-p) and CD44 (q-r) from Control and *Usp7* cKO intestine 1.5 years post-tamoxifen induction. Scale bars, 50 μm. High magnification images are shown in the insets. n=3 per group. (**s-u**) mRNA expression of the indicated genes was analyzed by qRT-PCR in crypts from 1.5 years old Control and *Usp7* cKO mice. Data are presented as fold change normalized to *hrpt1* (n=3 experimental replicates per condition). Error bars represent ± standard error. n=3 biological replicates per group. P-values were determined using the unpaired two-sided t-test (*P<0.05; **P<0.01; ***P<0.001).

Figure S3. Analysis of tumors derived from *Apc*^{min} and *Apc*^{min} *Usp7* cKO mice, related to Figure 3. (**a**) mRNA expression of the *Usp7* was analyzed by qRT-PCR in crypts from *Apc*^{min} Control and *Apc*^{min} *Usp7* cKO mice. Data are presented as fold change normalized to *hrpt1* (n=3 biological replicates per condition). Error bars represent ± standard error. P-values were determined using the unpaired two-sided t-test (***P<0.001). (**b**-**f**) Representative Caspase-3 (b), Edu (c) and SOX9 (e) stainings of tumors derived from the indicated genotypes 120 days after *Usp7* loss. Graphs in (d) and (f) indicate the number of Edu (d) or SOX9 (f) positive cells in 50 μm^2 of adenoma area. n=3 biological replicates per group. Scale bar, 100 μm. (**g**) Quantitation of the total number of tumors and tumor grades developed in the indicated mice at around 200 days after the first tamoxifen induction. Data are mean ± standard error. *Apc*^{min} control, n=7; *Apc*^{min} *Usp7* cKO, n=7. (**h**) Representative H&E staining from *Apc*^{min} control and *Apc*^{min} *Usp7* cKO mice showing enteritis present in the *Apc*^{het} *Usp7* cKO mice. Scale bar, 100 μm. (**i**) Representative CD68 stainings from *Apc*^{min} control

and *Apc*^{min} *Usp7* cKO mice collected at around 90 days and around 200 days. Scale bar, 100 μm.

Figure S4. Analysis of tumors derived from *Apc*^{het} and *Apc*^{het} *Usp7* cKO mice, related to Figure 4. (**a-f**) Immunostaining of *Apc*^{het} (Control) tumors (a, c, e) and *Apc*^{het} *Usp7* cKO (b, d, f) untransformed intestine 97 dpi using the indicated antibodies (n=3 biological replicates per group). Scale bars, 50 μm. (**g-h**) Quantitation of the total number of tumors (g) and tumor grades (h) developed in the indicated mice at around 200 days after the first tamoxifen induction. Data are mean ± standard error. *Apc*^{het} control, n=6; *Apc*^{het} *Usp7* cKO, n=6 biological replicates. (**i**) Representative H&E staining from *Apc*^{het} control and *Apc*^{het} *Usp7* cKO mice showing enteritis present in the *Apc*^{het} *Usp7* cKO mice. Scale bar, 100 μm. (**j**) Representative CD68 stainings from Apc^{het} control and *Apc*^{het} *Usp7* cKO mice collected at around 90 days and 200 days after induction. Scale bar, 100 μm. (**k**) Table depicting the number of mice that developed colitis and enteritis in the indicated genotypes and time. Scoring numbers are indicated as enteritis/colitis in the table. (**l**) Representative H&E stainings of the small intestines of the indicated genotypes. Scale bar, 2000 μm.

Figure S5. Inhibition of USP7 in human CRC patient-derived organoids suppresses cell growth and sensitizes 5-FU treatment, related to Figure 5. (**a**) Cell Titer Glo luciferase assay of the indicated PDOs treated with P22077 or vehicle (DMSO). Error bars represent ± standard error from n=3 independent experiments per each PDO donor. (**b**) Quantitation of the number of colonies formed in the indicated PDOs treated with DMSO or P22077 after 10 days. Data are mean ± standard error. At least n=3 per group from n=3 independent experiments per each PDO donor. (**c**) Cell Titer Glo luciferase assay of the indicated organoids treated with DMSO, P22077, 5-FU or both for 10 days. Error bars represent ± standard error from n=3 independent experiments per each PDO donor. (**d**) Representative images of colony formation assay of organoids from the organoids in (c). Scale bar, 1000 μm. (**e**) Quantitation of the number of colonies formed in the indicated PDOs in (d) treated with DMSO, P22077, 5-FU of both after 10 days. Data are mean ± standard error. At least n=3 experimental replicates per group from n=3 independent experiments per each PDO donor. P-values were determined using the unpaired two-sided t-test (*P<0.05; **P<0.01; ***P<0.001).

Figure S6. Inactivation of USP7 inhibits WNT signaling in APC-truncated PDO-derived xenografts *in vivo*, related to Figure 6. (**a**) Measurement of body weight of mice transplanted with PDO3 or PDO5 and treated with DMSO or P22077 inhibitor (30 mg/kg) at the end of the experiment (20 days) (n=4 biological replicates per condition). (**b**) Immunohistochemical analysis of PDO3- and PDO5-derived xenografts treated with DMSO or P22077 and stained with cleaved Caspase 3. Scale bar, 50 μm. (**c**) Tumor weight of PDO4 xenografts in DMSO control and P22077 treatment group (30 mg/kg) was measured at the end of treatment (21 days) (n=12 for DMSO, n=11 biological replicates for P22077). Error bars represent ± standard deviation. (**d**) Representative images of tumors derived from PDO4 at the end of treatment. (**e**) Tumor volumes of PDO4 treated with P22077 or vehicle at the indicated times. Error bars represent ± standard error. P-value was determined using the two-way ANOVA test (*P<0.05).

Supplemental Experimental Procedures

Real-time quantitative RT–PCR

RNA was extracted according to the manufacturer's instructions (Qiagen RNAeasy). cDNA was prepared using Maxima first strand cDNA synthesis kit with dsDNase (#1672, Thermo Scientific). Quantitative PCR detection was performed using iTaq SYBR Green Supermix (#172-5121, Bio Rad) using specific primers to: *hβ-ACTIN* F: 5' TTCTACAATGAGCTGCGTGTG 3' R: 5' GGGGTGTTGAAGGTCTCAAA 3'; *hCCND1*: F: 5' CTCCGCCTCTGGCATTTTGG 3' R: 5' TCTCCTTGCAGCTGCTTAG 3'; *hAXIN2*: F: 5' AGTGTGAGGTCCACGGAAAC 3' R: 5'´CTTCACACTGCGATGCATTT 3'; *hASCL2*: F: 5' GACCTGCGTACCTTGCTTTG 3' R: 5' CGCGCGATCACATTCTGTAA 3'; *hLGR5*: F: 5' ACTGCATCCTAAACTGCCCT 3' R: 5' TGTCCAGACGTAGGTTTGCT 3'; *mUsp7*: F: 5' TGCTGAATCTGACTCCACGT 3' R: 5' CCCAGTCGTTTTCCTTGTGG 3'; *mAxin2*: F:5' TCCAGAGAGAGATGCATCGC 3' R: 5' AGCCGCTCCTCCAGACTATG 3'; *mAscl2*: F: 5' AATGCAAGCTTGATGGACGG 3' R: 5' GGAAGCCCAAGTTTACCAGC 3'; *mLgr5*: F: 5' CATCAGGTCAATACCGGAGC 3' R: 5' TAATGTGCGAGGCACCATTC 3'; *mHrpt1*: F: 5'TCATGAAGGAGATGGGAGGC 3'; *mOlfm4*: F: 5' GACAGAGTGGAGCGCTTAGAG 3' R: 5' GCATCTCCTTCACTTCCAGC 3'; *mSox9*: F: 5' CTGGAGGCTGCTGAACGAGAG 3' R: 5' CGGCGGACCCTGAGATTGC 3'. After cDNA amplification (40 cycles), samples were normalized to β-actin (human) or *Hrpt1* (mouse), and data were expressed as mean ± SD.

Immunohistochemistry and Edu staining

For analysis of small intestine and colon by immunohistochemistry and Edu staining, tissues were fixed in 10% formalin and embedded in paraffin. For small intestinal tissues, same proximal part of the small intestine from all genotypes was used throughout the study. Immunohistochemistry was performed as described (30). The buffer used for antigen retrieval was citrate (SOX9, OLFM4, Keratin20 and Caspase-3). Edu staining was performed following manufacturer's instructions (C10338, Invitrogen). Mouse adenomas were graded by a pathologist by analyzing H&E stained sections as previously described (46). Colitis and enteritis were graded by analysis of H&E sections by a pathologist as described in Fig. 3g, Fig. S3g, Fig. 4g and Fig. S4i). The following parameters were histopathologically assessed and scored for small intestine enteritis assessment: a) epithelial injury (including villous atrophy in small intestine, 0-3), b) lamina propria inflammation (0-3), c) area (% section) affected (0-3) and d) markers of severe inflammation (including crypt abscesses, submucosal inflammation and/or ulceration, 0-3). The total possible score is thus out of 12.

RNAScope in situ hybridization

In situ hybridization (ISH) for Lgr5 was performed using the RNAScope FFPE assay kit (Advanced Cell Diagnostics) according to the manufacturer's instructions. Briefly, 4 μm formalin-fixed, paraffin-embedded tissue sections were pre-treated with heat and protease digestion before hybridization with the target probe. Then, an HRP-based signal amplification system was hybridized to the target probes (Lgr5, 312171) before color development with 3,30-diaminobenzidine tetrahydrochloride (DAB). 20 crypts from 3 mice per group were used to quantitate the number of Olfm4+ cells.

Mouse intestinal organoid culture

Organoids were established from freshly isolated wild-type (Control), Usp7 cKO, $Apc^{fl/fl}$ Control and $Apc^{fl/fl}$ Usp7 cKO small intestines. Tissues were incubated in cold PBS containing 2 mM EDTA for isolating epithelial crypts and culture as previously described (Sato, Vries et al. 2009) except that Matrigel was replaced with Cultrex BME, Type 2 RGF PathClear (Amsbio 3533-010-02). In brief, the organoid basal media contains EGF (Invitrogen PMG8043), Noggin and R-spondin (ENR) (5%). For $APC^{fl/fl}$ Control and $APC^{fl/fl}$ Usp7 cKO organoids, R-spondin was withdrawn from the media. The Rho kinase inhibitor Y-27632 (Sigma) was added to the culture when trypsinized. AKP colonic organoids were derived from Villin CreERT Apc, $Kras^{G12D}$ and $P53^{null}$ mice.

Human material and patient-derived organoid culture

Patient-derived organoids (PDOs) were derived from human CRC tissues that have been harvested during surgeries at the Royal Marsden Hospital (PDO1-2, REC reference 14/LO/1812), Guy's and St. Thomas' NHS Foundation Trust (PDO3, REC reference 12-EE-0493 and 18-EE-0025) and University College London Hospital (PDO4-5, REC reference 15/YH/0311) in accordance with ethical approval. Written informed consent was obtained. Intestinal samples were obtained from patients with colorectal cancer. Crypts were isolated from human intestinal tissue by incubating for 1 hour with chelation buffer (5.6 mM Na_2HPO_4, 8 mM KH_2PO_4, 96 mM NaCl, 1.6 mM KCl, 44 mM sucrose, 54.8 mM D-sorbitol, 0.5 M EDTA and 1M DTT at 4°C, and plated in drops of BME (Sato, Vries et al. 2009). After polymerization culture media was added. Human intestinal organoid media contains advanced DMEM/F12 medium (Invitrogen) including B27 (Invitrogen), nicotinamide (Sigma-Aldrich), N-acetylcysteine (Sigma-Aldrich), EGF (Invitrogen PMG8043), TGF-ß type I receptor inhibitor A83-01 (Tocris), P38 inhibitor SB202190 (Sigma-Aldrich), gastrin I (Sigma-Aldrich), WNT3a conditioned media (50% produced using stably transfected L cells), Noggin and R-spondin conditioned media. PDO1 and PDO2 were a kindly provided by Nicola Valeri's Lab. More details from these two patients can be found in their publication (Vlachogiannis, Hedayat et al. 2018).

Organoid colony formation assay

Organoids were trypsinized and counted. 2,000 single cells were seeded in BME per 48 wells and placed in a 37°C incubator to polymerize for 20 min. 250 µl of complete Organoid Growth media plus Y-27632 was then added and cultured for 6 days for mouse organoids and 10 days for human organoids. Number of spheres formed in each well was counted as plating efficiency. Experiments were performed in triplicate.

Cell Titer Glo

Organoids were trypsinized using TrypLE and filtered with a 20 µm cell strainer. 2000 single cells were seeded in BME per 48 well and placed in a 37°C incubator to polymerize for 20 minutes. 300 µl of complete Organoid Growth media was added for 10 days. The media was supplemented with 10 µM Y-27632 for 2 days after plating. Cell Titer Glo Luminiscent Cell viability assay (G7572, Promega) was used to assess viability of organoids. Experiments were performed at least three times with three triplicates each.

Western blot analysis

Organoids were treated with 10µM P22077 or vehicle (DMSO) for 3 days. At day 3, the organoids were pre-treated with 10µM MG132 proteasome inhibitor for 4h. Media

was aspirated from the wells and 1 ml of PBS containing 0.4 mg/ml of dispase was added to the wells and the plates were put back in the incubator for 20min. Then, this media was discarded and replaced by 1ml of cold PBS. The organoids drops were detached and centrifuged to 500 rcf, 5 min at 4ºC. Then the supernatant was discarded and the organoids lysed with 50 μl of lysis buffer containing 150 mM NaCl, 30 mM Tris (pH 7.5), 1 mM EDTA, 1% Triton X-100, 10% glycerol, 0.5 mM DTT plus protease and phosphatase inhibitors. Samples were centrifuged for 10 min at 12000 rpm at 4ºC and the supernatant was used for western blot analysis using the indicated antibodies.

Xenograft experiments
PDO3, PDO4 and PDO5 human organoids were subcutaneously injected into both flanks of 6-8-week-old NSG mice. The PDO-xenografts were allowed to grow 2-3 mm before randomizing the mice into a control group (DMSO) or P22077 (30 mg/kg) by intraperitoneal injection every day for 20 days. At the end of the experiments, all mice were culled. Tumors were collected, weighted, and photographed. All mice were housed in a pathogen-free environment and handled in strict accordance to institutional protocol.

References

Sato, T., R. G. Vries, H. J. Snippert, M. van de Wetering, N. Barker, D. E. Stange, J. H. van Es, A. Abo, P. Kujala, P. J. Peters and H. Clevers (2009). "Single Lgr5 stem cells build crypt-villus structures in vitro without a mesenchymal niche." Nature **459**(7244): 262-265.

Vlachogiannis, G., S. Hedayat, A. Vatsiou, Y. Jamin, J. Fernandez-Mateos, K. Khan, A. Lampis, K. Eason, I. Huntingford, R. Burke, M. Rata, D. M. Koh, N. Tunariu, D. Collins, S. Hulkki-Wilson, C. Ragulan, I. Spiteri, S. Y. Moorcraft, I. Chau, S. Rao, D. Watkins, N. Fotiadis, M. Bali, M. Darvish-Damavandi, H. Lote, Z. Eltahir, E. C. Smyth, R. Begum, P. A. Clarke, J. C. Hahne, M. Dowsett, J. de Bono, P. Workman, A. Sadanandam, M. Fassan, O. J. Sansom, S. Eccles, N. Starling, C. Braconi, A. Sottoriva, S. P. Robinson, D. Cunningham and N. Valeri (2018). "Patient-derived organoids model treatment response of metastatic gastrointestinal cancers." Science **359**(6378): 920-926.
